# Photocatalytic Degradation of Antibiotics Using Nanomaterials: Mechanisms, Applications, and Future Perspectives

**DOI:** 10.3390/nano16010049

**Published:** 2025-12-29

**Authors:** Jianwei Liu, Hongwei Ruan, Pengfei Duan, Peng Shao, Yang Zhou, Ying Wang, Yudi Chen, Zhiyong Yan, Yang Liu

**Affiliations:** 1 Beijing Engineering Research Center of Sustainable Urban Sewage System Construction and Risk Control, Beijing University of Civil Engineering and Architecture, Beijing 100044, China; liujianwei@bucea.edu.cn (J.L.); 2108300223016@stu.bucea.edu.cn (H.R.); 2 Beijing Academy of Science and Technology, Beijing 100089, China; 3 Institute of Analysis and Testing, Beijing Academy of Science and Technology (Beijing Center for Physical and Chemical Analysis), Beijing 100089, China; 4 Department of Chemistry, Key Lab of Bioorganic Phosphorus Chemistry and Chemical Biology of Ministry of Education, Tsinghua University, Beijing 100084, China

**Keywords:** photocatalytic degradation, antibiotic pollution, nanomaterials, artificial intelligence, ecological environment

## Abstract

Widespread antibiotic residues in aquatic environments pose escalating threats to ecological stability and human health, highlighting the urgent demand for effective remediation strategies. In recent years, photocatalytic technology based on advanced nanomaterials has emerged as a sustainable and efficient strategy for antibiotic degradation, enabling the effective utilization of solar energy for environmental remediation. This review provides an in-depth discussion of six representative categories of photocatalytic nanomaterials that have demonstrated remarkable performance in antibiotic degradation, including metal oxide-based systems with defect engineering and hollow architectures, bismuth-based semiconductors with narrow band gaps and heterojunction designs, silver-based plasmonic composites with enhanced light harvesting, metal–organic frameworks (MOFs) featuring tunable porosity and hybrid interfaces, carbon-based materials such as g-C_3_N_4_ and biochar that facilitate charge transfer and adsorption, and emerging MXene–semiconductor hybrids exhibiting exceptional conductivity and interfacial activity. The photocatalytic performance of these nanomaterials is compared in terms of degradation efficiency, recyclability, and visible-light response to evaluate their suitability for antibiotic degradation. Beyond parent compound removal, we emphasize transformation products, mineralization, and post-treatment toxicity evolution as critical metrics for assessing true detoxification and environmental risk. In addition, the incorporation of artificial intelligence into photocatalyst design, mechanistic modeling, and process optimization is highlighted as a promising direction for accelerating material innovation and advancing toward scalable, safe, and sustainable photocatalytic applications.

## 1. Introduction

The ubiquitous presence of antibiotics in aquatic environments has emerged as a pressing global environmental challenge, threatening both ecological balance and human health [1]. Designed to combat bacterial infections, these pharmaceutical compounds are increasingly detected in surface water, groundwater, and even drinking water, largely because conventional wastewater treatment processes exhibit limited ability to fully remove them [2]. Their continuous discharge into the environment promotes the development and proliferation of antibiotic-resistant bacteria and resistance genes, thereby diminishing the efficacy of antibiotic therapies and constituting a serious public health crisis. Furthermore, even at low concentrations, antibiotics can exert toxic effects on non-target aquatic organisms and pose potential risks to human health through chronic exposure [3]. Thus, there is an urgent need for effective and sustainable technologies to eliminate or degrade antibiotics from contaminated water sources [4,5].

Traditional water treatment methods, such as physical adsorption, flocculation, and chemical oxidation, face inherent limitations, including incomplete removal, generation of toxic byproducts, high energy demand, and the production of secondary waste requiring further treatment [6]. Advanced oxidation processes (AOPs), which involve the generation of highly reactive species such as hydroxyl radicals (•OH) and superoxide radicals (${•O}_{2}^{-}$), have shown great potential for degrading persistent organic pollutants, including antibiotics [7]. Among these processes, heterogeneous photocatalysis stands out for its ability to utilize solar or artificial light to convert pollutants into less harmful substances, mainly carbon dioxide and water, with the added benefits of low cost, high efficiency, and minimal secondary pollution [8]. It is well established that the efficiency of photocatalysis is heavily dependent on the properties of the photocatalyst, especially its capacity to absorb light, generate electron–hole pairs, and facilitate charge separation and transfer to react with adsorbed pollutants and water/oxygen to form reactive oxygen species (ROS) [9]. In this context, nanomaterials, with their large surface areas, unique electronic band structures, and quantum size effects, have emerged as highly promising candidates in the design of efficient photocatalysts for environmental remediation [10].

Recent advances in nanotechnology have facilitated the design and fabrication of a wide range of nanomaterial-based photocatalysts, such as metal oxides, bismuth-based semiconductors, silver-based composites, MOFs, carbon-based materials, and emerging MXene hybrids [4,11,12]. However, while existing reviews have extensively documented the synthesis and laboratory-scale efficiency of these materials, a critical gap remains in bridging the disconnect between fundamental material design and practical environmental application. Most current literature tends to focus heavily on degradation rates in ultrapure water, often overlooking the complexity of real-world water matrices (e.g., pH variability, coexisting ions, and natural organic matter) that drastically influence catalyst performance and stability. Furthermore, the potential formation of intermediate byproducts that may be more toxic than the parent antibiotics is frequently under-addressed, necessitating a shift from simple “pollutant removal” to comprehensive “toxicity elimination”. Moreover, the rapid emergence of Artificial Intelligence (AI) offers a transformative opportunity to overcome the trial-and-error limitations of traditional catalyst development. Unlike previous reviews that treat AI as a disconnected or supplementary topic, this review posits AI as a central pillar for the future of photocatalysis, from accelerating material screening and predicting structure-activity relationships to optimizing complex reactor parameters.

**Figure 1:**
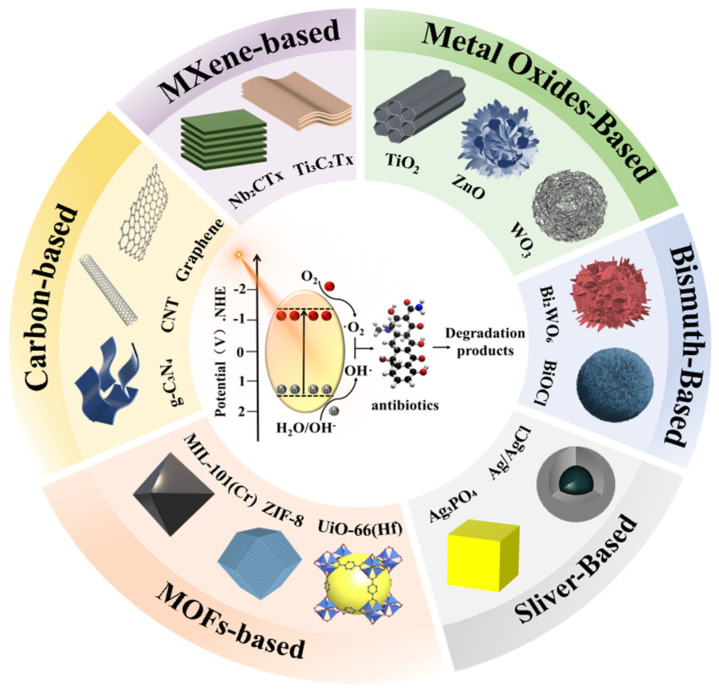
Overview of advanced photocatalytic nanomaterials for antibiotic degradation.

Therefore, this review provides a comprehensive and critical analysis of state-of-the-art photocatalytic nanomaterials, distinguishing itself by integrating three key dimensions: (i) A systematic evaluation of six major classes of photocatalysts (Figure 1), focusing on how specific nanostructures (e.g., defects, heterojunctions) drive performance; (ii) An in-depth discussion on the challenges of scalability, matrix effects, and toxicity evolution, emphasizing the gap between laboratory proof-of-concept and real-world detoxification; and (iii) A holistic perspective on AI-driven strategies that are reshaping the roadmap for discovering next-generation photocatalysts. By connecting material engineering with environmental toxicity assessment and data-driven science, this review aims to provide a clear pathway toward scalable, safe, and sustainable photocatalytic water treatment technologies.

## 2. Antibiotics in Aquatic Systems: Physicochemical Characteristics, Transformation, and Ecological Risks

### 2.1. Chemical Structure and Physicochemical Properties of Antibiotics

As emerging contaminants, antibiotics exert adverse effects that extend beyond their clinical application in humans and animals to impact ecosystems. Based on differences in chemical structures and characteristics, antibiotics are mainly classified into β-lactams (e.g., penicillin, cephalosporins), tetracyclines (e.g., tetracycline (TC), doxycycline), fluoroquinolones (e.g., ciprofloxacin (CIP), norfloxacin (NOR)), and sulfonamides [13]. While these antibiotics can kill or inhibit pathogenic microorganisms, including bacteria and fungi in human and animal hosts, their use may also lead to side effects, such as persistent toxicity to aquatic organisms, allergic reactions, and even organ damage in humans [14]. However, from a photocatalytic perspective, the significance of these classifications lies not just in their biological activity, but in their specific structure-activity relationships that dictate degradation efficiency. The molecular structure determines both the “anchoring groups” required for catalyst adsorption and the “reactive sites” susceptible to radical attack.

The structural diversity of antibiotics directly governs their environmental behavior. Tetracyclines, characterized by a four-ring system containing hydroxyl, keto, and dimethylamino groups, exhibit strong adsorption onto biochar and metal oxides due to their hydrophilicity and ability to form hydrogen bonds [15]. Crucially, the keto-enol functional groups in TCs can act as ligands to form inner-sphere surface complexes with metal sites via ligand-to-metal charge transfer. This specific interaction significantly lowers the activation energy for interfacial electron transfer, often serving as a primary pathway for direct hole (h^+^) oxidation. Furthermore, the high electron density of the phenolic ring makes it a preferential target for electrophilic attack by •OH [16]. In contrast, fluoroquinolones, with their aromatic quinolone core and carboxyl groups, preferentially adsorb onto hydrophobic materials such as sludge-derived biochar [17]. Recent theoretical calculations indicate that the piperazine ring in FQs usually possesses the highest Fukui index, making it the most fragile site for oxidative cleavage during photocatalysis [18]. However, structural modifications, such as halogenation at the C-8 position (e.g., lomefloxacin), can prolong environmental persistence by resisting enzymatic and photolytic breakdown [19]. The zwitterionic nature of certain FQs (e.g., CIP) further complicates removal, as it allows dual interactions with both hydrophilic and hydrophobic matrices depending on the solution pH. Regarding other classes, it is understood that β-lactams are rapidly degraded through hydrolysis of the β-lactam ring under neutral or alkaline conditions; however, derivatives with bulky substituents (e.g., third-generation tetracyclines like tigecycline) exhibit enhanced resistance to degradation. In photocatalytic systems, the cleavage of the strained β-lactam ring is often accelerated by ${•O}_{2}^{-}$ attack. Sulfonamides, which harbor sulfonamide (-SO_2_NH_2_) and aromatic amine groups, typically exhibit moderate persistence; while the S-N bond is vulnerable to attack, their degradation intermediates may still retain antimicrobial activity, necessitating deep mineralization to ensure detoxification.

Most antibiotics possess stable physicochemical properties, such as acidity, alkalinity, and electrostatic properties, which are determined by their molecular composition. Antibiotics are typically ionic polar organic compounds containing functional groups like hydroxyl (-OH), amine (-NR^3+^), and carboxyl (-COOH), along with their corresponding acid dissociation constants (pK_a_). Depending on the pH conditions, antibiotics can exist in various forms including cationic, amphoteric, neutral, or anionic forms [20]. This speciation is critical for the “electrostatic gatekeeping” effect in photocatalysis. It has been established that dissociation sites change at different pH values: when pH ≤ pK_a1_, the antibiotic exists as a cation, enabling adsorption onto negatively charged surfaces via electrostatic attraction. Conversely, when the pH exceeds the Point of Zero Charge of the photocatalyst and the pK_a_ of the antibiotic, electrostatic repulsion hinders the approach of pollutant molecules to the active sites, thereby suppressing the reaction rate [21]. Thus, the pH of the solution plays a significant role in affecting not only the generation of ROS and dissociation of surface charges but also the protonation state of antibiotic molecules The basic properties of antibiotics are summarized in Table 1.

### 2.2. Transformation Products, Detoxification, and Environmental Risk

In photocatalytic antibiotic removal, it is increasingly recognized that “disappearance of the parent molecule” does not necessarily equate to detoxification, because partial oxidation often yields transformation products (TPs) that can retain bioactivity or even exhibit higher ecotoxicity than the pristine antibiotic. Therefore, an environmentally meaningful assessment should couple kinetic removal with mechanistic elucidation of TP formation (e.g., hydroxylation, dealkylation, ring-opening, and defluorination pathways) and a clear accounting of mineralization (TOC/COD) rather than relying solely on C/C0. For example, detailed TP identification during antibiotic phototransformation has been performed by combining kinetics with intermediate tracking, enabling a more defensible discussion of pathway reliability and controlling factors such as water matrix components and catalyst surface chemistry [22,23]. Importantly, TP analysis should be integrated with bioassays to verify true detoxification, because post-treatment solutions may contain reactive aromatic intermediates and short-chain oxygenates whose health and ecological impacts are not captured by routine concentration-based metrics. Evidence from pharmaceutical photodegradation illustrates this “double-edged” nature of photocatalysis: acetaminophen can yield intermediates that are potentially valorisable (e.g., 1,2,4-benzenetriol) under certain photocatalytic systems [24,25,26], yet hazardous intermediates such as p-nitrophenol have also been detected depending on catalyst and irradiation conditions, underscoring that pathway steering is central to risk control [27]. Beyond TP-derived risks, the catalyst itself may introduce environmental burdens via photo/chemical corrosion and consequent leaching of metal ions or nanoscale fragments; such “nanotoxicity” concerns motivate the use of greener synthesis, protective surface coatings, and rigorous post-reaction characterization to ensure that improved reactivity does not trade off against stability and safety [28,29]. Collectively, a robust “TP–detoxification–risk” narrative should explicitly link (i) TP fingerprints and dominant pathways, (ii) mineralization and detoxification endpoints, and (iii) catalyst stability/leaching, so that photocatalysis is evaluated not only for antibiotic degradation, but also for verified risk reduction in realistic water-treatment contexts.

## 3. Principle and Fundamental Mechanism of Photocatalytic Degradation of Antibiotics

AOPs have emerged as an innovative solution for wastewater purification, characterized by mild conditions, exceptional degradation performance, and reliable process stability. These systems leverage diverse mechanisms such as electromagnetic wave-assisted decomposition (ultrasound/microwave), acoustic cavitation, ozone-based oxidation, low-temperature plasma techniques, iron-catalyzed redox processes (Fenton variants), electrochemical treatments, and light-driven catalytic reactions to produce reactive intermediates, including •OH, ${•O}_{2}^{-}$, and electron vacancies (h^+^) [30]. These oxidizing agents possess robust redox potential capable of mineralizing recalcitrant organic compounds through combined oxidation-reduction pathways. Photocatalytic processes are characterized by a high degree of mineralization efficiency, achieving comprehensive degradation via radical-mediated chain reactions [31]. This green technology demonstrates environmental compatibility, cost-effectiveness, and operational simplicity, holding significant potential for application in the elimination of antibiotic compounds from water. However, it should be emphasized that the apparent removal of antibiotics does not necessarily indicate complete mineralization, as partial oxidation may lead to the formation of transformation products with altered environmental behavior and biological effects.

**Figure 2:**
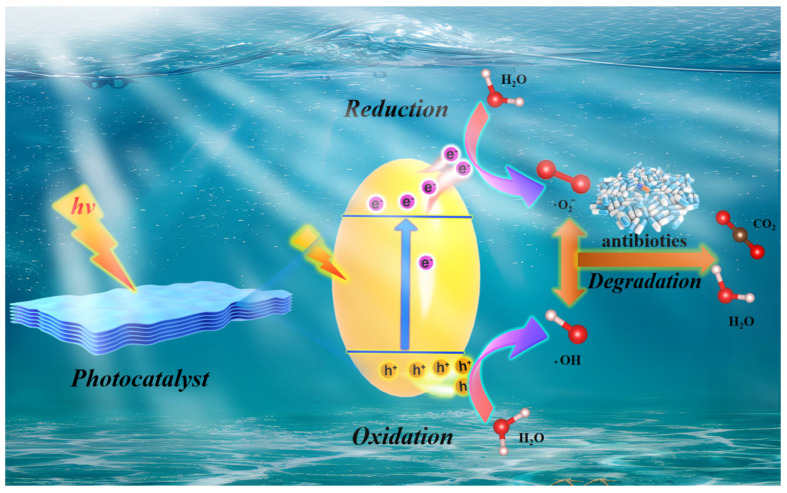
The general photocatalytic mechanism of antibiotic degradation by photocatalysts.

Photocatalytic reactions are now understood to occur in three main stages. Initially, when the energy of light (including ultraviolet, visible, and infrared light) meets or exceeds the semiconductor bandgap (Eg), electrons in the photocatalytic material are excited from the valence band (VB) to the conduction band (CB), forming photogenerated electrons (e^−^), which possess reducing properties. Corresponding photogenerated holes (h^+^) with oxidizing abilities are created in the VB. At this point, a highly active photogenerated electron–hole pair, interconnected by Coulomb forces, is formed in the semiconductor [32]. Due to the discontinuity in the semiconductor’s energy bands, these photogenerated electron–hole pairs separate under the influence of an electric field and diffuse toward the surface of the semiconductor. However, not all diffused electrons and holes will participate in the catalytic reaction. Due to their short lifespans, photogenerated electrons and holes are prone to recombination through diffusion, a process known as bulk recombination. Even if they reach the semiconductor surface, surface recombination may occur due to their mutual attraction [33]. Finally, electrons and holes that successfully reach the surface react with various compounds through redox reactions. At the catalyst surface, electrons may reduce O_2_ to produce ${•O}_{2}^{-}$, or further react to form •OH. Holes can react with OH^−^ in water or oxidize adsorbed hydroxyl groups on the catalyst surface, also generating •OH [34]. Due to the potent oxidative activity of holes and •OH radicals, and to a lesser extent, ${•O}_{2}^{-}$ radicals, these species can chemically interact with the target pollutants, producing intermediate products that contribute to the degradation of pollutants. Because these processes occur competitively and simultaneously, the relative contribution of different reactive species strongly depends on band-edge positions, surface chemistry, defect states, and solution conditions such as pH and dissolved oxygen. Figure 2 illustrates the mechanism of photocatalytic degradation of pollutants.

Research has established that, in photocatalytic decomposition systems, the principal oxidizing agents responsible for contaminant transformation are light-induced h^+^ and •OH formed through valence band charge carriers interacting with surface-bound hydroxyl groups or water molecules [35]. While ${•O}_{2}^{-}$ participate in the reaction network, their contribution is less pronounced due to diminished oxidative capacity. The degradation mechanism is formalized through a series of chemical Equations (1)–(5), delineating the sequential electron transfer processes and radical-mediated breakdown pathways.

Semiconductor + hν → e^−^ + h (1)

(2) $$e^{-}\;+\;O_{2}\;\to {•O}_{2}^{-}$$

(3) $${•O}_{2}^{-}\;+\;H_{2}O\;\to \;•\mathrm{OH}$$

h^+^ + OH^−^ → •OH (4)

(5) $$h^{+}/{•O}_{2}^{-}/•\mathrm{OH}\;+\;\mathrm{Pollutant}\;\to \;{\mathrm{CO}}_{2}\;+\;H_{2}O$$

The primary goal involves converting hazardous contaminants into benign substances while pursuing complete transformation into inorganic constituents (such as carbon dioxide, water, and mineral salts) when technologically feasible. Critical technical challenges include suppressing both bulk-phase electron–hole pair annihilation and interfacial charge recombination phenomena. Nanomaterials, by virtue of their unique physicochemical properties, offer robust solutions to these challenges. Their high specific surface area provides abundant active sites and enhances pollutant adsorption. Quantum size effects and tailorable electronic band structures enable the modulation of bandgap energies for improved visible-light utilization [36]. Furthermore, nanoscale dimensions facilitate shorter charge migration distances to the surface, reducing bulk recombination losses. Engineered nanostructures (e.g., hollow spheres, heterojunctions, defect engineering) can further optimize light harvesting, charge separation dynamics, and interfacial reactions [37]. Therefore, the rational design and engineering of advanced photocatalytic nanomaterials have emerged as the central strategy to enhance the efficiency and applicability of photocatalytic antibiotic degradation.

## 4. Key Photocatalytic Nanomaterials Used in Antibiotic Degradation

### 4.1. Metal Oxide-Based Photocatalysts

Metal oxide-based photocatalysts are widely employed alone or in combination with other materials to accelerate the degradation of organic pollutants such as insecticides, dyes, and polycyclic aromatic hydrocarbons [38]. The past decade has witnessed a burgeoning interest in leveraging metal oxide-containing photocatalysts specifically for the degradation of antibiotics. These materials effectively absorb both visible (Vis) and ultraviolet (UV) light and exhibit high biocompatibility, safety, and stability under various environmental conditions. However, their practical application is hindered by their wide bandgap and the rapid recombination of photogenerated electron–hole pairs, which limits the formation of strong oxidizing species such as •OH, ${•O}_{2}^{-}$, and singlet oxygen (^1^O_2_) under light irradiation [39]. Therefore, strategic modifications or the development of composite systems is essential to improve electron–hole separation and fully realize their photocatalytic potential.

In recent years, spherical metal oxides have been widely employed as efficient heterogeneous photocatalysts for antibiotic degradation, owing to their distinctive features such as high specific surface area, excellent permeability, and pronounced porosity. In a study by Ding et al. [40], who fabricated a hollow-spherical CuFe_2_O_4_ catalyst enriched with oxygen vacancies (HS-CuFe_2_O_4-σ_) via a one-step hydrothermal approach for heterogeneous Fenton-like degradation of CIP. The hollow architecture provides a larger accessible surface and facilitates mass transport, while oxygen vacancies act as key redox-active sites that promote electron transfer and accelerate H_2_O_2_ activation toward radical generation. Notably, HS-CuFe_2_O_4-σ_ achieved complete CIP removal within 30 min over a relatively broad pH window (6.5–9.0), and the apparent rate constant at neutral pH was markedly enhanced compared with pristine CuFe_2_O_4_. Beyond demonstrating high removal efficiency, this work also highlights an important design principle for metal oxides in antibiotic treatment: vacancy-rich surfaces and nanoconfined hollow structures can cooperatively lower activation barriers for oxidant activation and pollutant oxidation, thereby mitigating the pH sensitivity that commonly restricts Fenton-like systems. In addition, Xu et al. [41] synthesized a Z-scheme Cu_2_O/Bi/Bi_2_MoO_6_ heterojunction featuring Bi-decorated hollow microflower spheres. This material leveraged surface plasmon resonance (SPR) from Bi nanoparticles to simultaneously oxidize sulfadiazine and reduce Ni (II), while the hollow structure enhanced light harvesting and localized electric fields. The Z-scheme configuration further promoted charge separation. Besides vacancy engineering, multicomponent oxide assemblies have been developed to extend solar responsiveness and strengthen interfacial charge utilization. Zhang et al. [42] prepared SiO_2_@Fe_2_O_3_@TiO_2_ hollow spheres (SFT) using an SiO_2_ template strategy, where ultrafine Fe_2_O_3_ domains are integrated with TiO_2_ to promote charge separation and surface redox reactions. Importantly, SFT was evaluated under natural sunlight in addition to simulated irradiation, which better reflects practical solar-driven scenarios. Under sunny conditions, TC could be completely decomposed within 80 min, and near-complete TC degradation was still achieved under cloudy weather after extended irradiation; ENR was also rapidly removed (complete degradation reported within 80 min). These findings emphasize that rationally designed oxide composites can maintain high activity under fluctuating solar intensity; however, they also imply that cross-study comparison of “rate constants” should be made cautiously, because weather, photon flux, reactor geometry, and adsorption contributions can substantially alter apparent kinetics even when the same catalyst is used.

**Figure 3:**
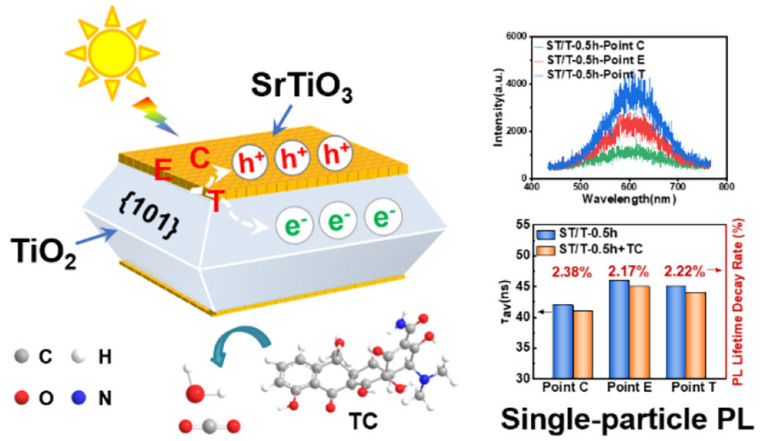
Single-particle charge-transfer behavior of SrTiO_3_/TiO_2_ heterointerfaces for photocatalytic tetracycline degradation [45].

To address the inherent limitations of single-component metal oxide photocatalysts, advanced composite systems that integrate distinct functionalities have emerged as a dominant strategy for enhancing antibiotic degradation efficiency. While TiO_2_ remains a cornerstone material due to its superior UV-driven activity in the anatase phase, its wide bandgap (~3.2 eV) and rapid charge recombination necessitate advanced design strategies. To overcome these drawbacks, hybrid architectures combining TiO_2_ with carbonaceous supports or hierarchical structures have shown great promise. Abdullah et al. [43] synthesized activated carbon-TiO_2_ composites (ACT-X) via hydrothermal methods. Among these, the ACT-4 photocatalyst demonstrated the highest photocatalytic degradation (99.6%) of ceftriaxone sodium through synergistic adsorption–photocatalysis. The conductive carbon matrix enhanced visible-light absorption and suppressed electron–hole recombination, showcasing the efficacy of hybrid designs. To bridge performance enhancement with mechanistic and application-oriented outcomes, single-atom or quasi-atomic cocatalyst strategies have recently been introduced into oxide photocatalysis. Wang et al. [44] constructed a biochar-supported single-atom iron cocatalyst coupled with anatase TiO_2_ (SA-Fe@TiO_2_), in which isolated Fe sites are anchored on nitrogen-doped biochar and interfaced with TiO_2_ This architecture was shown to facilitate charge-carrier separation/transfer and to increase the apparent rate constant for SMX degradation by 4.3 times relative to TiO_2_, while also exhibiting an enhanced capacity for inactivating antibiotic-resistance-related genetic risks. Beyond pollutant disappearance, the authors further considered mineralization and broader applicability: substantial TOC elimination was reported during SMX treatment, and immobilized SA-Fe@TiO_2_ films enabled rapid degradation of multiple antibiotics (five distinct antibiotics degraded within 20 min), highlighting a pathway toward more deployable systems. Such results are highly relevant to the recurring concern in antibiotic photocatalysis that “degradation” does not necessarily equal “detoxification”: catalyst designs that concurrently improve oxidation capacity and suppress harmful by-products/secondary risks (e.g., ARG propagation) should be preferentially emphasized in future evaluations. Beyond conventional ensemble measurements, single-particle spectroscopy has recently been used to elucidate charge dynamics in metal oxide heterojunctions for antibiotic degradation. Li et al. constructed a facet-selective SrTiO_3_/TiO_2_ epitaxial heterojunction, in which SrTiO_3_ mesocrystals are topotactically grown on the {001} facets of decahedral TiO_2_, enabling photogenerated electrons to migrate to the TiO_2_ {101} facets while holes accumulate in SrTiO_3_ (Figure 3). By combining this interfacial charge-transfer scheme with position-resolved PL intensity and lifetime mapping of individual particles, they showed that the epitaxial heterointerface substantially prolongs carrier lifetimes and suppresses PL decay during in situ tetracycline degradation, directly linking ordered oxide interfaces with enhanced photocatalytic performance toward antibiotics [45].

Beyond TiO_2_, iron-based oxides, such as α-Fe_2_O_3_ and Fe_3_O_4_, have attracted significant interest due to their visible-light absorption (bandgap 2.1–2.3 eV) and magnetic recoverability. However, their low charge separation efficiency remains a challenge. Recent advances have addressed this through composite design. In this regard, Yilmaz et al. [46] designed TiO_2_@ Fe_3_O_4_@C-NFs composites, where magnetic Fe_3_O_4_ nanospheres modified with TiO_2_ nanoparticles and carbon nanofibers achieved 80–100% degradation of antibiotics and azo dyes under UV light. The carbon nanofibers enhanced electron transfer, while Fe_3_O_4_ enabled easy magnetic separation, addressing both stability and recyclability issues.

Cerium oxide (CeO_2_) has attracted significant interest given its oxygen vacancy-driven redox properties. Lu et al. [47] synthesized CeO_2_ nanorods with abundant oxygen vacancies, which achieved 89.35% TC degradation under visible light. The vacancies acted as electron traps, prolonging carrier lifetimes and enabling efficient Ce^3+^/Ce^4+^ cycling, thereby outperforming CeO_2_ nanomaterials with other morphologies (such as nanocubes and nanosheets). Zinc oxide (ZnO), despite its high electron mobility, suffers from photocorrosion in aqueous environments. Recent studies have integrated ZnO with carbon-based materials to enhance its stability. For instance, Roy et al. [48] synthesized ferrocene-functionalized rGO-ZnO nanocomposites, achieving >95% removal of CIP and SMX. The rGO framework provided conductive pathways for charge separation, while its 3D structure enhanced light absorption, demonstrating the versatility of metal oxide-carbon hybrids in improving both stability and performance. Another effective route is to couple metal oxides with carbonaceous components and engineer built-in electric fields through step-scheme heterojunctions. Wang et al. [49] synthesized an MOF-derived N-doped ZnO carbon skeleton and grew hierarchical Bi_2_MoO_6_ nanosheets in situ to form a 3D layered S-scheme heterojunction (N-ZnO/C@Bi_2_MoO_6_). By combining structural advantages (highly open 3D framework) with defect features (oxygen vacancies) and an S-scheme charge-transfer pathway, the composite achieved strongly improved kinetics for SMX photodegradation under visible light. The pseudo-first-order kinetic constant reached 0.022 min^−1^, reported as 10× that of the MOF-derived ZnO component and 27.5× that of pristine Bi_2_MoO_6_; high SMX removal was achieved within 60 min in the authors’ tests. Importantly, this study also devoted attention to charge-transfer driving forces and degradation pathways, including mechanistic interpretation of S-scheme behavior and pathway analysis supported by computation, which is crucial for moving the field beyond empirical “activity boosting” toward more predictive design rules. The photocatalysis characteristics of antibiotics by metal oxide-based photocatalysts are summarized in Table 2.

### 4.2. Bismuth-Based Photocatalysts

Bismuth, a metallic element from Group 5 of Period 6 in the periodic table, has an atomic electron configuration of 6s^2^6p^3^ and typically exists in the form of Bi^3+^. Notably, the lone-pair distortion of the Bi 6s orbital in bismuth-based complex oxides can result in the overlap between the O 2p and Bi 6s orbitals in the valence band, which contributes to a reduced band gap and enhances the mobility of photoinduced charges, thereby improving the material’s visible light absorption performance [50]. Furthermore, the Bi^5+^ valence state, once the 6s orbital is empty, exhibits effective visible light absorption. Among the most extensively studied bismuth-based photocatalysts are Bi_2_O_3_, BiVO_4_, Bi_2_PO_4_, Bi_2_WO_6_, and bismuth-based halogen oxides (BiOX, where X = Cl, Br, or I) [51].

**Figure 4:**
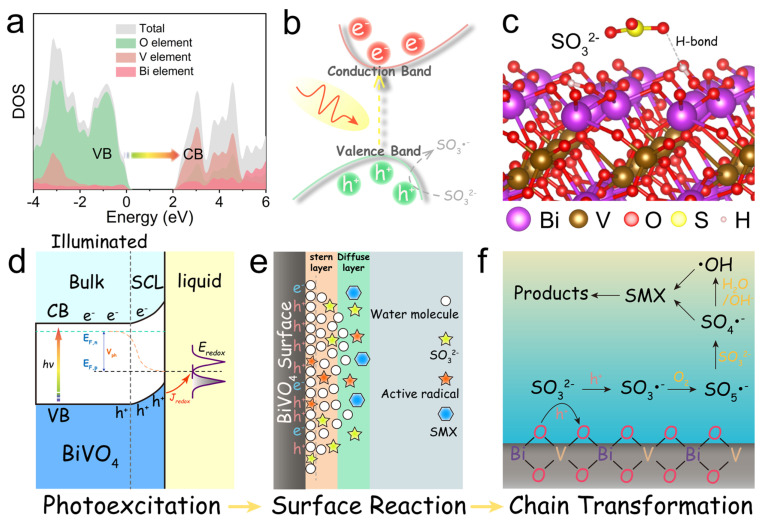
Electronic structure and solid–liquid interface reaction mechanism of BiVO_4_. (**a**) DOS calculation for BiVO_4_; (**b**) the proposed photocatalytic decomposition process of SO_3_^2−^ on the BiVO4; (**c**) adsorption of monodentate SO_3_^2−^ on the BiVO_4_ with (110) facet; (**d**) quasistatic energy profile and charge transfer pathways of BiVO_4_ under continuous illumination in contact with the aqueous solution. J_redox_ is the target charge transfer from the valence band to the redox reagent, SCL is space charge layer, *V_ph_* is the open-circuit photovoltage. *E_F_*_,*n*_ and *E_F_*_,*p*_ are the quasi-Fermi levels of electrons and holes under illumination, respectively; (**e**) model of the double-layer structure of BiVO_4_ in contact with the aqueous solution under equilibrium conditions; (**f**) transformation pathways of major species in Na_2_SO_3_/BiVO_4_ [55].

Recent advancements in bismuth-based photocatalysts underscore the pivotal roles of heterojunction engineering and defect modulation in enhancing antibiotic degradation. Notably, the Cd_0_._5_Zn_0_._5_S/carbon dots/Bi_2_WO_6_ (CZS/CDs/BWO) S-scheme heterojunction, synthesized via hydrothermal and solvothermal methods, utilizes carbon dots as interfacial electron mediators to suppress charge recombination, achieving a 6.4-fold higher TC degradation rate than pristine Bi_2_WO_6_ by preserving redox potentials through directional charge transfer [52]. Employing an alternative approach, a Z-scheme Au@TiO_2_/Bi_2_WO_6_ heterojunction was fabricated through reverse micelle sol–gel and hydrothermal techniques, combines plasmonic Au nanoparticles with Bi_2_WO_6_ nanosheets. This design broadened visible-light absorption via Au’s surface plasmon resonance and enhanced charge separation, enabling 96.9% SMX degradation within 75 min [53]. Beyond heterojunction configurations, defect engineering has emerged as an equally pivotal strategy. For instance, TaON/Bi_2_WO_6_ nanofibers with OVs, prepared by electrospinning and in situ growth, harness OVs as electron traps to suppress recombination while maintaining redox capacity via an S-scheme pathway, resulting in a 2.8-fold TC degradation improvement over bare Bi_2_WO_6_ [54]. While the rational design of heterojunctions and defects optimizes light absorption and bulk charge separation, the ultimate photocatalytic degradation efficiency is often governed by the interfacial charge transfer kinetics at the solid–liquid interface. For many bismuth-based photocatalysts that operate via non-radical pathways (e.g., direct hole oxidation), the reaction is confined to the immediate catalyst surface. Organic pollutant molecules, especially those with low polarity, can be effectively excluded from this reactive zone due to surface solvation and the formation of an electrical double layer, leading to severely limited kinetics despite thermodynamically favorable conditions. This critical barrier was systematically investigated in a study focusing on BiVO_4_. The researchers revealed that although BiVO_4_ has a high valence band potential, it could not degrade SMX directly. They identified a “surface solvation-induced inactivation” mechanism, where a hydrophilic surface and structured water layer prevent organic molecules from reaching the photo-generated holes [55]. To overcome this, they introduced sulfite (SO_3_^2−^) as a redox mediator. Sulfite, with its higher adsorption affinity and lower ionization potential, can penetrate the compact layer, be oxidized by holes, and initiate a chain reaction to generate highly oxidizing sulfate radicals (SO_4_•^−^) that diffuse out to degrade pollutants (Figure 4). This work highlights that interface engineering, such as employing judicious redox mediators, is a crucial strategy to complement material-level modifications for achieving high degradation performance with bismuth-based photocatalysts.

For many Bi-based oxides/oxyhalides, the intrinsic limitation is still the rapid recombination of photoinduced electrons and holes and the insufficient antibiotic, catalyst contact under realistic matrices. Thus, defect modulation (especially oxygen vacancies) and morphology control (ultrathin/porous structures) are widely adopted to simultaneously enhance light harvesting, adsorption, active-site density, and charge separation. Using TC as a representative antibiotic. Gao et al. [56] reported oxygen-vacancy–rich ultrathin porous Bi_2_WO_6_ nanosheets (V_O_-rich BWO), where the nanosheets were only 1.83 nm thick and exhibited abundant porous channels for mass transfer and interfacial reaction acceleration. The adsorption behavior of TC on V_O_-rich BWO followed pseudo-second-order kinetics (with R^2^ ≈ 0.9993), and the rate-controlling steps were attributed to diffusion and pore-filling processes, highlighting that well-designed porosity is not merely “surface area increase” but can reshape the adsorption–photocatalysis coupling mode in Bi-based systems. Under visible light, V_O_-rich BWO achieved 95.12% TC removal within 100 min (10 mg/L TC,0.2 g/L catalyst), and showed stable performance over multiple cycles; superoxide radicals (${•O}_{2}^{-}$) were identified as the dominant reactive species.

Another mainstream route to upgrade Bi-based photocatalysts is to build heterojunctions that enforce directional charge migration while preserving strong redox potentials. However, heterojunction effectiveness is highly sensitive to interface quality, band alignment, and built-in electric fields; consequently, the same “strategy label” (e.g., vacancy engineering or heterojunction construction) can produce widely scattered efficiencies across reports if interface and kinetics are not carefully controlled. In a recent study, a novel dual S-scheme heterojunction photocatalyst, β-Bi_2_O_3_/NiAl-LDH/α-Bi_2_O_3_, was designed and fabricated. This composite was constructed through the phase transformation of Bi_2_O_3_ and coupling with layered double hydroxides (LDHs). The dual heterojunction was driven by internal electric fields (IEFs) and favorable band bending at the interface, significantly enhancing charge carrier separation and redox potential. Mechanistic investigations, corroborated by XPS and DFT calculations, revealed that electrons could migrate from the LDH to Bi_2_O_3_, generating internal electric fields and facilitating directional charge transfer. This charge transfer process promoted efficient photodegradation via synergistic radical pathways (${•O}_{2}^{-}$, •OH, h^+^) [57].

Furthermore, coupling bismuth-based materials with carbonaceous or metallic components has been shown to enhance stability and reactivity. In this regard, a study revealed that surface oxygen vacancies in Nd-doped BiVO_4_ enabled efficient chlorite activation, converting ${\mathrm{ClO}}_{2}^{-}$ to reactive ClO_2_ for 96% cephalexin degradation under visible light [58]. Importantly, DFT calculations and in situ DRIFTS suggest that the OV-introduced surface -OH serves as the Bronsted acidic center for chlorite adsorption. Likewise, reduced graphene oxide (rGO)/Bi_4_O_5_Br_2_ nanocomposites achieved 97.6% CIP removal through synergistic adsorption and a multi-radical (${•O}_{2}^{-}$, •OH, h^+^) pathway [59]. Similarly, BiVO_4_/O-g-C_3_N_4_ heterojunctions were engineered with interfacial chemical bonds and Ovs, achieving 99.8% TC removal via synergistic redox pathways [60]. For BiOX (BiOBr in particular), a practical bottleneck is that pristine BiOBr often suffers from high carrier recombination and limited affinity toward certain organics; moreover, the preparation of complex multi-phase heterojunctions can be synthetically demanding. Using an in situ self-reduction route, Jiang et al. constructed a regenerable (Bi) BiOBr/rGO composite, in which metallic Bi nanoparticles are “perfectly lattice-matched” with the BiOBr matrix and can strengthen visible-light utilization through a surface plasmon resonance (SPR) effect, while rGO serves as an adsorptive support and fast electron-transfer network. Experimentally, the incorporation of Bi and rGO broadened visible-light absorption, lowered PL intensity (indicating suppressed recombination), and improved photocurrent/EIS behavior, collectively supporting more efficient charge separation and interfacial electron transport. Importantly, the study went beyond typical batch tests: the catalyst showed robustness in a continuous photocatalytic operation for 50 h, and in a continuous-flow column configuration almost 100% TC removal could be maintained for 10 h using TC-spiked river water, highlighting a device-oriented pathway for translating Bi-based powders toward application-relevant reactor modes [10]. In another study, surface oxygen vacancies in BiCrO_4_/g-C_3_N_4_ composites facilitated 92.5% levofloxacin (LEV) degradation within 120 min under LED light irradiation, where vacancies acted as electron highways to enhance interfacial charge transfer, outperforming vacancy-free counterparts by 2.3-fold [61].

To address the long-standing practical issue of nanopowder recovery and to better bridge laboratory degradation to engineered water-treatment units, another promising direction is immobilizing Bi-based photocatalysts on flexible/porous scaffolds that also offer adsorption enrichment. Using carbonized eggshell membrane (CEM) as a bio-derived support, Zhou et al. prepared a CQDs/BiOCl/CEM composite and achieved a clear adsorption–photodegradation synergy: under 10 mg/L TC and 0.5 g/L catalyst loading, the optimized sample delivered 98% total TC removal by combining 30 min dark adsorption and 30 min visible-light degradation [62]. Beyond concentration removal, the work also evaluated mineralization-related performance in a more complex matrix: in industrial wastewater the COD removal reached 73.1% after 120 min, substantially higher than pristine BiOCl (49.9%), suggesting that structural/charge modulation and support-assisted adsorption can improve not only decolorization/degradation but also deeper oxidation outcomes in realistic water samples. Given that post-treatment toxicity can be driven by transformation intermediates, embedding such “COD/mineralization-aware” comparisons into Bi-based photocatalyst discussions is particularly valuable for a review that aims to connect performance metrics with environmental safety.

Moreover, support materials play a crucial role in enhancing the performance of bismuth-based photocatalysts. Recent studies have highlighted the effectiveness of 3D porous sponges and zeolites as supports. For instance, Gao et al. [63] developed a 3D lignosulfonate-composited poly(vinyl formal) (PVF) sponge with a ternary photocatalyst, BiVO_4_/polyaniline (PANI)/Ag (PLS-BiVO_4_/PANI/Ag), for fluoroquinolone degradation. In this system, the lignosulfonate enhanced the sponge’s 3D structure, improving its adsorption capabilities for fluoroquinolones, while the uniformly distributed photocatalysts facilitated efficient photodegradation. Under near-neutral pH, over 90% removal was achieved in batch tests, with stable removal rates of 80% over 1800 min in continuous treatment. Similarly, zeolites have been used as supports to improve photocatalytic performance. Shabani et al. [64] synthesized Bi_2_Sn_2_O_7_-C_3_N_4_/Y (BSO-BCN/Y) photocatalysts supported on zeolites. Their findings indicate that the zeolite support enhanced the distribution of active phases, increased active sites, and improved the separation of electron–hole pairs, significantly boosting both adsorption and photocatalytic activity. Furthermore, novel BiOBr/CsXWO_3_@SiO_2_ aerogels further utilized pore-expansion templates to achieve near-complete antibiotic removal through adsorption/self-heating photothermal synergy [65]. These results emphasize the importance of selecting suitable support materials to optimize the efficiency of bismuth-based photocatalysts. The photocatalytic performance of various bismuth-based photocatalysts for antibiotic degradation is summarized in Table 3.

### 4.3. Silver-Based Photocatalysts

Silver (Ag) nanoparticles and silver-based compounds are frequently incorporated into photocatalytic systems to enhance performance, primarily due to the unique properties of silver, such as its strong light absorption, high conductivity, surface plasmon resonance property, narrow bandgap, outstanding quantum efficiency, photosensitive property, and photo-electrochemical features [66]. Among these, Ag_3_PO_4_, Ag_3_VO_4_, and Ag/AgX (X = Cl, Br, I) systems stand out for their visible-light responsiveness and redox capabilities. These silver-based photo-catalysts exhibit remarkable effectiveness in degrading antibiotics, primarily due to their ability to limit the recombination rate of electron–hole pairs and their wide spectrum of light absorption [67].

Silver-based semiconductors exhibit exceptional photooxidation potential under visible light. However, their practical application is limited by rapid photo-corrosion and electron–hole recombination. To address these dual challenges, constructing Z-scheme heterojunctions has proven effective. Cai et al. [68] developed an inorganic–organic Z-scheme photocatalyst by anchoring Ag_3_PO_4_ onto self-assembled PDI supramolecular rods (PDIsm) via electrostatic interactions. This improvement was attributed to the promoted charge separation/transfer enabled by the Z-scheme architecture, which also helps mitigate photocorrosion by reducing undesired electron accumulation on Ag_3_PO_4_. Building on this, Fan et al. [69] integrated 0D Ag_3_PO_4_ with 2D NiAl-LDH via a room-temperature precipitation strategy, establishing a Z-scheme system that exhibited 61.6-fold higher TC degradation rates than NiAl-LDH alone. Beyond conventional heterojunctions, structural engineering has been employed to further enhance performance. For instance, novel Ag/AgBr/AgI@SiO_2_ composite aerogels with controlled pore structures were developed, improving TC removal through a synergistic adsorption–photocatalysis effect. By optimizing pore volume and specific surface area via N,N-dimethylacetamide expansion, these aerogels achieved efficient ${•O}_{2}^{-}$-mediated degradation across a broad pH range (2–10) while maintaining high recyclability [70]. Liu et al. [71] developed Ag nanocluster-modified Ag_3_PO_4_ containing silver vacancies (Ag/Ag_3_PO_4_-VAg) via an in situ reduction method, significantly enhancing SMX degradation activity and stability. The Ag nanoclusters provided a localized surface plasmon resonance (LSPR) effect, while Ag vacancies improved electron trapping and water adsorption capacity, enabling complete degradation of 100 mL 20 mg/L SMX within 15 min with a remarkable rate constant of 0.306 min^−1^, which was 17 times that of pure Ag_3_PO_4_. The enhanced performance was attributed to improved charge separation and strong LSPR-induced local electric fields.

**Figure 5:**
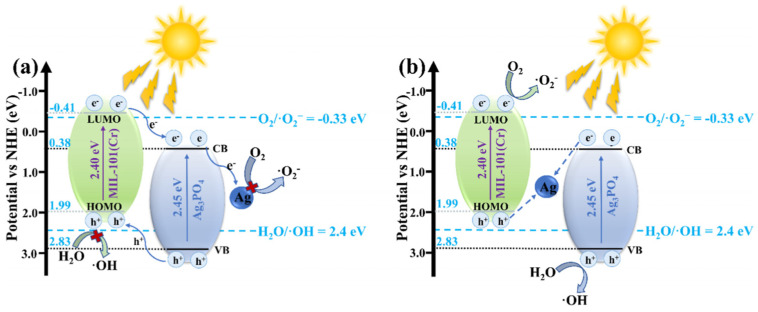
(**a**) Traditional type II heterojunction model and (**b**) Z-type heterojunction model of AAM [72].

In this context, Wang et al. [72] designed an all-solid-state Z-scheme Ag/Ag_3_PO_4_/MIL-101(Cr) (AAM) heterostructure, which provides a representative example of how band engineering and metallic Ag bridges can be exploited to reconcile activity and stability in silver-based systems. On the basis of Tauc plots, Mott–Schottky analysis and VB-XPS, they showed that a conventional type-II band alignment between Ag_3_PO_4_ (E_CB ≈ +0.38 V vs. NHE) and MIL-101(Cr) (E_LUMO ≈ −0.41 V vs. NHE) would place photogenerated electrons on the CB of Ag_3_PO_4_ and holes on the HOMO of MIL-101(Cr), which is thermodynamically insufficient to drive O_2_/${•O}_{2}^{-}$ and H_2_O/•OH conversions, in conflict with ESR and radical-trapping results. Consequently, an indirect Z-scheme pathway mediated by interfacial metallic Ag was proposed. As illustrated by the band-structure models of AAM (Figure 5), photoexcited electrons in the CB of Ag_3_PO_4_ and holes in the HOMO of MIL-101(Cr) are funneled to Ag nanoparticles, where they recombine, leaving highly reducing electrons in MIL-101(Cr) to generate ${•O}_{2}^{-}$ and strongly oxidizing holes in the VB of Ag_3_PO_4_ to form •OH. This Z-scheme configuration markedly enhances spatial charge separation while preserving strong redox potentials on both components, thereby underpinning the superior TC degradation efficiency and photostability of the Ag/Ag_3_PO_4_/MIL-101(Cr) photocatalyst compared with its single counterparts.

In addition, the integration of plasmonic Ag nanoparticles has emerged as a successful strategy to amplify light harvesting and charge carrier dynamics in photocatalytic systems. Specifically, Liu et al. [73] designed Ag/Ag_3_PO_4_/C_3_N_5_ S-scheme heterojunctions, where the LSPR from Ag nanoparticles broadened visible-light absorption, achieving 28.38-fold higher LEV degradation than C_3_N_5_. Similarly, another practical direction is building multifunctional composites that integrate adsorption enrichment, photocatalysis, and convenient recovery. Liao et al. [74] prepared a magnetic Fe_3_O_4_@mTiO_2_@Ag@GO composite via a microwave-assisted route, combining (i) Fe_3_O_4_ for magnetic recyclability, (ii) TiO_2_ as a stable photocatalytic scaffold, (iii) Ag nanoparticles to broaden visible-light response through LSPR, and (iv) GO to enhance adsorption and interfacial electron transport. For NOR removal, Fe_3_O_4_@mTiO_2_@Ag@GO delivered a photodegradation rate 4.6× that of Fe_3_O_4_@mTiO_2_ and 1.4× that of Fe_3_O_4_@mTiO_2_@Ag, underscoring the synergistic contribution of GO-assisted adsorption/charge transfer on top of Ag plasmonic enhancement. Furthermore, a synergistic approach combining photocatalysis with other methods has also proven effective. For instance, a 3D Bombax-structured Ag_3_PO_4_/carbon nanotube sponges demonstrated 90% TC degradation under combined ultrasound–visible light irradiation. The ultrasound-induced cavitation effect (100 W) enhanced charge transfer efficiency, contributing over 50% of the total degradation kinetics while reducing energy demand [75]. Crucially, the synergistic effect of Ag NPs and GO is crucial for achieving significant enhancements in photocatalytic performance.

Recent advancements have further demonstrated that integrating silver catalysts with tailored carriers improves both stability and scalability. Importantly, recent review emphasized the gap between powder photocatalyst demonstrations and real-world deployment (recovery, continuous operation, matrix effects). In this context, immobilization into films is a valuable Ag-based implementation pathway. Vanlalhmingmawia et al. [4] developed Clay/TiO_2_/Ag^0^ nanocomposite films and demonstrated simultaneous degradation of TC and SMZ, achieving 72.4% TC and 58.3% SMZ removal within 60 min, while maintaining >90% stability over six cycles, which features that directly address catalyst retrieval and reuse. In parallel, Negoescu et al. [76] harnessed activated carbon (AC) as a co-surfactant for TiO_2_-based photocatalysts: the Ag/Ag_2_O/TiO_2_-AC composite leveraged AC hydrophobicity and large surface area to synergize adsorption and photocatalysis for CIP removal. Collectively, these carrier-integrated systems transcend mere activity enhancement, offering scalable solutions for practical implementation. The photocatalysis characteristics of antibiotics by silver-based photocatalysts are presented in Table 4.

### 4.4. MOFs-Based Photocatalysts

In recent years, MOFs, a new class of functional inorganic-organic hybrid materials, have experienced rapid development. They are porous, crystalline structures formed by the coordination of metal ions or small metal ion clusters (nodes) with organic ligands (linkers) [77]. MOFs offer several advantages over traditional photocatalytic semiconductors, making them promising for applications in antibiotic adsorption and degradation in water. First, their porosity of MOFs and their open frame structure facilitate the diffusion of degraded substances to the active sites of the catalyst. Secondly, MOFs have an extremely high specific surface area and adjustable porosity. Such properties are suitable for constructing composite catalytic materials with other active substances. Finally, the spectral response range of MOFs can be well regulated by introducing functional groups [78,79]. These properties make MOFs promising as effective materials for antibiotic adsorption and photocatalysis in water. Recent efforts have focused on combining MOFs with other materials to bolster catalytic efficiency, yielding MOF composite photocatalysts, which underscores the viability of this approach.

The establishment of heterojunctions significantly improves the photocatalytic efficiency of MOFs by enhancing the separation of photo-generated electron–hole pairs, increasing light absorption, and providing more reactive sites, which collectively boost photocatalytic performance. A representative approach is to couple MOFs with visible-light-active semiconductors to form heterojunctions that promote charge separation while retaining the adsorption capability of MOFs. Li et al. [80] fabricated an S-scheme MIL-101(Fe)/Bi_2_WO_6_ heterostructure. The integration of MIL-101(Fe) octahedrons enlarged the surface area and introduced oxygen vacancies, facilitating charge carrier separation. This engineered heterojunction exhibited a 10.5-fold increase in the photocatalytic activity toward TC degradation compared to the individual components. Importantly, mineralization was also evaluated, with a measurable TOC reduction reported within the same reaction period, indicating that the process went beyond mere transformation. Similarly, Su et al. [81] constructed direct Z-scheme Bi_2_MoO_6_/UiO-66-NH_2_ heterojunctions with flower-like morphology, enabling 100% ofloxacin (OFL) and 96% CIP degradation within 90 min via synergistic •OH/h^+^/${•O}_{2}^{-}$ pathways while reducing toxicity. Ternary heterojunctions further optimize performance: Liu et al. [82] designed a Z-scheme NH_2_-MIL-125(Ti)/Ti_3_C_2_ QDs/ZnIn_2_S_4_ system, wherein MXene quantum dots accelerated interfacial electron transfer, achieving 96% TC degradation in 50 min. Nano-heterojunction engineering has also demonstrated efficacy. Cao et al. [77] synthesized SnS_2_@UiO-66 via crystal regrowth, enlarging active sites and achieving 90% TC degradation in 75 min (5.1× faster than UiO-66) under visible light.

Metal doping represents another effective approach to modify MOF photocatalysts. Current evidence suggests that doped metals can facilitate electron transfer through metal-to-metal electron transfer (MMCT). A typical example is Cu incorporation into UiO-66 to generate oxygen-vacancy-related defects and broaden light utilization [83]. The modified MOF exhibited substantially improved adsorption toward CIP: about half of the target antibiotic could be captured, corresponding to an adsorption capacity on the order of 53.7 mg g^−1^, which was far higher than that of pristine UiO-66. Under full-spectrum Xe lamp irradiation, the optimized Cu-doped UiO-66 removed 93% of CIP within 1 h with a kinetic constant of 0.03257 min^−1^, and the overall photocatalytic performance was reported to be several-fold higher than that of the parent UiO-66. Mechanistically, the enhancement was attributed to the synergistic role of (i) adsorption enrichment in the porous framework, (ii) defect/metal-assisted electron transfer, and (iii) improved separation of photogenerated carriers, which collectively increased ROS formation and accelerated CIP breakdown. In addition, cyclic tests indicated that the catalyst retained activity after repeated use, with only a slight decline attributed to partial occupation of adsorption sites by residual organics. Similarly, Du et al. [84] utilized Co-MOFs as precursors to fabricate nitrogen-rich Co-doped C_3_N_5_ (Co-C_3_N_5_). The highly dispersed Co sites narrowed the bandgap to ∼1.20 eV, enabling complete degradation of 30 mg/L chlortetracycline hydrochloride (TCH) within 6 min under a 50 W LED lamp via peroxymonosulfate (PMS) activation. Li et al. [14] demonstrated that sulfidation of Zn/In-MOFs yielded self-assembled hollow microtubular Zn-In-S structures (e.g., ZnS/ZnIn_2_S_4_). The Zn dopant was crucial for forming a direct Z-scheme heterojunction, which enhanced charge separation and boosted reactive oxidant generation (•OH, ${•O}_{2}^{-}$, h^+^), achieving >90% TC degradation within 1 h under solar light (kinetic constant: 0.0379 min^−1^). Beyond elemental doping, Liu et al. [85] constructed a Z-scheme Ti-MOF/Ag/NiFeLDH photocatalyst by depositing plasmonic Ag nanoparticles onto NH_2_-MIL-125(Ti)/NiFe LDH hybrids. The Ag nanoparticles acted as electron bridges, facilitating interfacial charge transfer and significantly enhancing visible-light degradation of LEV (92% in 70 min) primarily via ${•O}_{2}^{-}$ and •OH radicals, while maintaining >90% efficiency after 5 cycles.

**Figure 6:**
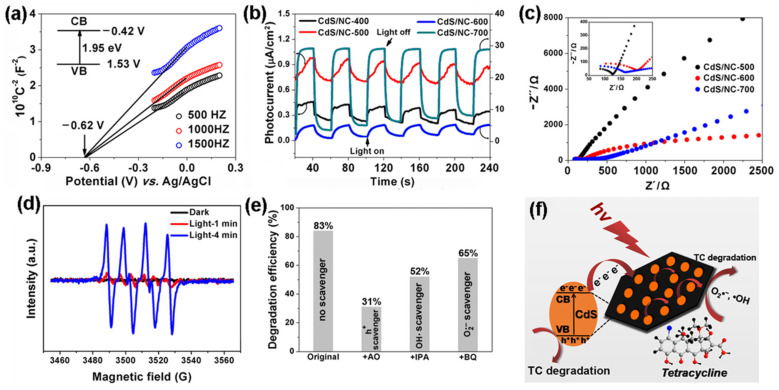
(**a**) Mott–Schottky plots for CdS/NC-500. (Inset) Energy diagram of the CB and VB levels. (**b**) Transient photocurrent plots for CdS/NC-T. (**c**) Electrochemical impedance spectra of CdS/NC-T. (**d**) DMPO–${•O}_{2}^{-}$ spin-trapping ESR spectra for CdS/NC-500. (**e**) Photocatalytic efficiency of CdS/NC-500 toward TC degradation with exposure to various scavengers. (**f**) Proposed mechanism of photocatalytic degradation of antibiotics for CdS/NC-500 [86].

MOFs can also be used as sacrificial templates/precursors to construct semiconductor–carbon composites with boosted charge transport. Cao et al. [86] prepared CdS/nitrogen-doped carbon (CdS/NC-T) photocatalysts by in situ carbonization of a cadmium MOF, and the optimized CdS/NC-500 sample showed markedly enhanced visible-light degradation of tetracycline compared with pristine CdS and low-temperature analogs. Electrochemical analyses, including Mott–Schottky plots, transient photocurrent responses and EIS measurements (Figure 6), confirmed efficient separation and interfacial transfer of photogenerated carriers in CdS/NC-500. Combined with DMPO–ESR and scavenger experiments, which identified h^+^, •OH and ${•O}_{2}^{-}$ as the dominant reactive species, they proposed a mechanism in which the conductive N-doped carbon matrix acts as an electron highway and adsorption platform, thereby accelerating redox reactions during antibiotic photodegradation. Morphological control can also effectively improve the photodegradation efficiency of antibiotics within MOFs. In this regard, Yu et al. [87] immobilized UiO-66-NH_2_/BiOBr heterojunctions on macroscale carbon fiber cloth via solvothermal and dip-coating methods. This unique macroscopic morphology enabled strong antibiotic adsorption (65.4% LEV) and facilitated recyclability, achieving 92.2% visible-light degradation within 120 min while maintaining stable efficiency over 4 cycles. Similarly, Zhang et al. [88] designed pomegranate-shaped ZnO@ZIF-8 through a template-directed synthesis, in which ZIF-8 was grown in situ on petal-shaped ZnO. This hierarchical structure provided high specific surface area to enrich TC, resulting in 91% degradation within 50 min under visible light and 100% activity retention after 5 cycles. Fang et al. [89] further demonstrated that sulfidation of MIL-68-In precursors yielded hollow In_2_S_3_ nanorods with invaginated hexagonal morphology. The hollow structure enhanced light absorption and electron–hole separation, enabling efficient degradation of both dyes and TC hydrochloride. To address the practical challenge of catalyst recovery and to bridge the gap between powder photocatalysts and engineering deployment, MOF-based photocatalysts have also been integrated into structured or immobilized architectures. A notable route is to assemble MOF-containing hybrids with conductive carbon and semiconductor components, then fabricate monolithic or 3D-structured photocatalysts for easier handling. In a WO_3_–UiO-66@reduced graphene oxide (rGO) system, coupling UiO-66 with WO3 and rGO improved both adsorption and charge transport, while the structured (3D) configuration further increased accessibility and facilitated reuse [90]. Such results highlight that structural engineering (e.g., 3D configuration) can be an effective complement to compositional engineering, enabling both high activity and improved operational convenience.

Catalyst recovery has long been a limiting factor for the practical application of photocatalysis. To address this, researchers have explored various methods for recovering MOF-based materials, including the preparation of magnetic materials to assist in separation. For instance, Zhang et al. [91] fabricated a novel MOF-derived ZnFe_2_O_4_/Fe_2_O_3_ perforated nanotube photocatalyst via direct calcination of MIL-88B/Zn, forming an intimate Z-scheme heterojunction with hierarchical porous architecture. The optimized sample of ZnFe_2_O_4_/Fe_2_O_3_ perforated nanotubes achieved a high CIP degradation efficiency of 96.5% under simulated light irradiation. The degradation of CIP under these conditions follows two main pathways. The first pathway involves hydroxylation and piperazine ring cleavage of CIP, leading to various intermediates that are eventually mineralized into CO_2_ and H_2_O. The second pathway involves the electrophilic addition of •OH, resulting in intermediates such as IP8 (*m*/*z* = 348) and IP9 (*m*/*z* = 304). In addition, the photocatalyst exhibited magnetic behavior with a saturation magnetization of 0.44 emu/g, enabling rapid magnetic separation and maintaining 92.6% of its activity after five consecutive cycles, confirming its excellent reusability. Similarly, Suo et al. [92] engineered a spindle-shaped ZnO/ZnFe_2_O_4_ Z-scheme heterojunction from MIL-88A(Fe)@Zn, achieving 86.3% TC hydrochloride degradation under visible light. Its intrinsic magnetism (coercivity: 108 Oe) facilitated effortless recovery from tap water, maintaining 83.74% efficiency after five uses. Overall, these studies confirm that magnetic MOF-derived photocatalysts enhance recyclability without compromising performance, addressing a critical barrier for scalable wastewater treatment. The photocatalysis characteristics of MOF-based photocatalysts for antibiotic degradation are summarized in Table 5.

### 4.5. Carbon-Based Photocatalyst

Carbon-based photocatalysts, including graphene derivatives, biochar, and graphitic carbon nitride (g-C_3_N_4_ or CN), have demonstrated significant potential in photocatalytic antibiotic degradation. Key advantages include inexpensive synthesis, high SSA, a well-developed and adjustable network of micropores, mesopores, and macropores, and a large number of oxygen-containing functional groups, all contributing to their efficacy [93,94].

Graphene and reduced graphene oxide (rGO) are widely employed as electron mediators in heterojunction systems to suppress charge recombination and broaden light absorption [95]. For instance, a Z-scheme Cu_2_O/rGO/BiVO_4_ composite, synthesized via a two-step solvothermal method, achieved 96% TC degradation under visible light. The in situ growth of Cu_2_O and BiVO_4_ on rGO nanosheets created intimate interfacial contact, enabling rGO to act as an electron bridge for directional charge transfer while preserving the high redox potentials of individual components [96]. Similarly, rGO-ZnS-CuS nanocomposites, prepared by a surfactant-free microwave-assisted approach, degraded 93% OFL via dominant ${•O}_{2}^{-}$ and •OH radical pathways. The microwave-induced rapid nucleation ensured uniform dispersion of ZnS-CuS nanoparticles on rGO, enhancing light absorption and exposing active sites for radical generation [97]. Graphene and its derivatives are typically incorporated as conductive 2D networks to promote interfacial electron migration and to increase accessible surface area, thereby improving both adsorption and photocatalytic kinetics. A recent study employed cerium-doped leaf-like CdS coupled with ultrathin nitrogen-doped rGO (Ce-CdS/N-rGO). The preparation process of Ce-CdS/N-rGO composites involves multiple steps, including ultrasonic dispersion of GO, followed by hydrothermal synthesis to form Ce-CdS, and further modification with nitrogen-doped reduced graphene oxide (N-rGO). The Ce^3+^/Ce^4+^ redox pairs act as electron capture sites within the CdS matrix, facilitating the interfacial charge transfer, which is enhanced by the strong interaction between Ce-CdS and N-rGO, as confirmed by DFT calculations. This synergistic effect of doping and interface engineering, combined with the enhanced adsorption capacity provided by N-rGO, resulted in a remarkably high TC removal efficiency of 94.5% under visible light, primarily driven by ${•O}_{2}^{-}$ generated through the efficient interfacial charge transfer. The mass spectrometry (MS) results further suggested stepwise oxidation/reduction and bond-cleavage processes, ultimately producing smaller molecules and partial mineralization products [98].

It is well established that biochar, a sustainable carbon matrix derived from biomass, enhances photocatalytic performance by improving adsorption capacity and providing defect-rich active sites. For example, TiO_2_ nanoparticles were uniformly immobilized onto pomelo-peel-derived biochar via a liquid-phase deposition followed by pyrolysis, forming a dense and crystalline composite structure. The optimal sample exhibited a low Raman ID/IG ratio of 0.05, indicating well-restored graphitic domains that facilitated electron transport. Combined with the hierarchical porosity of the biochar matrix, this architecture significantly enhanced charge separation and pollutant adsorption, ultimately achieving a TC degradation rate constant of 0.021 min^−1^ under simulated solar light, and maintained near 80% removal efficiency after five cycles, indicating acceptable reusability (with the slight loss mainly attributed to catalyst loss during recovery) [99]. Similarly, CoFe-LDH supported on petrochemical sludge biochar was prepared through a hydrothermal co-precipitation method. The biochar’s high conductivity (Raman D/G ratio of 0.05) enabled rapid electron transfer, achieving complete SMX removal within 100 min, while the LDH’s layered structure provided abundant active sites for hydroxyl radical generation [100]. Biochar composites further facilitate the integration of noble metals. For instance, Ag@biochar-rGO nanohybrids, synthesized using marine algae *Trentepohlia* sp. as a green reductant, demonstrated dual functionality by degrading rifampicin via •OH while reducing Cr(VI) under sunlight, leveraging rGO electron shuttling and Ag’s localized surface plasmon resonance [101]. In addition, a pinecone-derived biochar, when used in a CaIn_2_S_4_-ZnO/Biochar S-scheme system fabricated via hydrothermal-wet impregnation, achieved >95% TC degradation. The biochar’s oxygen-containing functional groups (e.g., C-OH) anchored the heterojunction, stabilizing charge transfer pathways and enhancing H_2_O_2_ production for radical-driven degradation [102].

**Figure 7:**
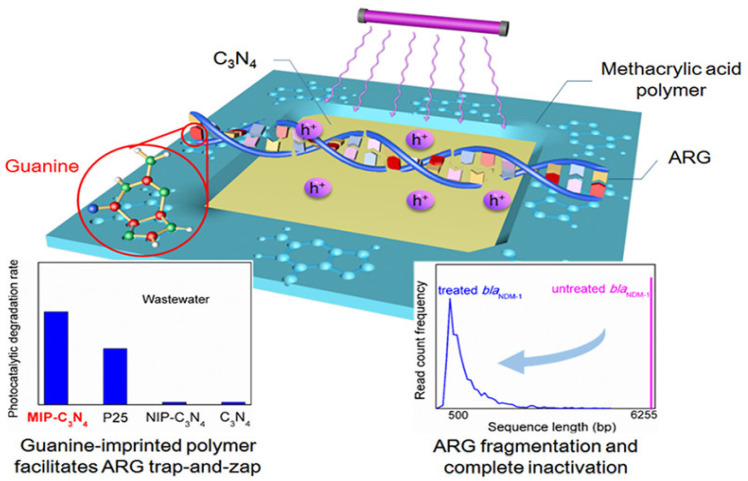
Schematic illustration of molecularly imprinted g-C_3_N_4_ (MIP-C_3_N_4_) for selective adsorption and photocatalytic degradation of extracellular antibiotic resistance genes [108].

Graphitic carbon nitride (g-C_3_N_4_) has emerged as a research hotspot in carbon-based photocatalysis due to its visible-light responsiveness (bandgap ~2.7 eV), chemical stability, and low cost. Significant improvements in charge separation efficiency and light absorption range have been achieved through morphology engineering, elemental doping, and heterojunction construction. For instance, oxygen-doped biochar-modified g-C_3_N_4_ (A-CN), synthesized by calcining hydroxyl-rich aloe fiber-derived precursors, demonstrated a reduced bandgap (2.2 eV vs. pristine 2.7 eV) and achieved 95% TC degradation with 88% mineralization within 1 h under visible light. This performance originated from hole-mediated deamidation and ROS-driven oxidative ring-opening pathways, enabling deep antibiotic mineralization [103]. Moreover, heterojunction designs with transition metal carbides (e.g., Co-Mo_2_C/g-C_3_N_4_) enhanced interfacial electric fields, accelerating charge separation. The composite exhibited a TC degradation rate three times higher than pristine g-C_3_N_4_, attributed to enhanced electron density in Mo 3d orbitals and Co-catalyzed oxygen reduction reactions [104]. As a metal-free visible-light-responsive photocatalyst, g-C_3_N_4_ is often upgraded via defect engineering (e.g., N vacancies) and/or single-atom modulation to improve light absorption, charge separation, and ROS production, Zhang et al. [105] constructed a Mo single-atom anchored N-vacancy tubular g-C_3_N_4_ (Mo/Nv-TCN) photocatalyst through a dual optimization strategy combining morphological control and defect engineering. The introduction of nitrogen vacancies and atomically dispersed Mo enabled the formation of a stable Mo–2C/2N coordination structure, which acted as a bridge for directional charge transfer. This structural refinement led to a narrowed bandgap and enhanced visible-light absorption capacity. Under visible-light irradiation, the optimized Mo/Nv-TCN system achieved 94.45% TC degradation within 60 min, with an apparent rate constant 4.46 times that of pristine g-C_3_N_4_. Mechanistic investigations further revealed that the photocatalytic degradation involved multiple ROS, including ${•O}_{2}^{-}$, h^+^, and ^1^O_2_, with Mo sites serving as the main active centers. Beyond “pure” carbon nitride, constructing 2D/2D heterostructures is another effective carbon-based route to improve adsorption and accelerate interfacial charge transfer, a 2D/2D S-type heterojunction has been developed by intercalating ultrathin g-C_3_N_4_ into NH_4_V_4_O_10_ nanosheet. This architecture, exemplified by the 50-CNNS/NH_4_V_4_O_10_ composite, achieved an impressive 92% removal rate of CIP under simulated sunlight, demonstrating excellent stability and photocatalytic performance. Confirming the enhanced photocatalytic degradation when compared to the separate components. The improved catalytic efficiency was attributed to the efficient charge separation, interfacial electron transport, and increased surface adsorption enabled by the exposed NH_4_^+^ groups, which promote the adsorption of CIP molecules via hydrogen bonding with fluoride (F^−^). From an environmental-safety standpoint, the authors also evaluated V leaching by ICP: the dissolved vanadium after recycled reaction was 0.0668 mg/L, which they noted was below the cited drinking-water limit (0.1 mg/L), suggesting manageable secondary-pollution risk under their conditions [106]. Zhang et al. [107] developed xB-PCN, a phosphorus and boron co-doped hollow tubular g-C_3_N_4_, which exhibited significantly enhanced photocatalytic activity for CIP degradation under visible light. The introduction of p-n heterojunctions within a single g-C_3_N_4_ facilitated efficient charge separation, resulting in 87.56% CIP removal in 60 min and excellent stability after 5 cycles with only a 2.59% reduction in efficiency. Beyond the removal of antibiotic molecules, g-C_3_N_4_ can also be engineered to mitigate the dissemination of antibiotic resistance. Yuan et al. [108] fabricated molecularly imprinted graphitic carbon nitride (MIP-C_3_N_4_) nanosheets, in which guanine was used as a template to create specific recognition cavities for a plasmid-encoded extracellular antibiotic resistance gene (bla_NDM-1_) in secondary effluent. As schematically illustrated in Figure 7, the guanine-imprinted sites endow MIP-C_3_N_4_ with a “trap-and-zap” function: selective adsorption enriches eARGs near the photocatalyst surface, allowing photogenerated holes to efficiently fragment the DNA while minimizing scavenging by background organic matter. Consequently, photocatalytic removal of bla_NDM-1_ in real wastewater (k = 0.111 ± 0.028 min^−1^) was approximately 37-fold faster than with pristine g-C_3_N_4_ (k = 0.003 ± 0.001 min^−1^), and the resulting short DNA fragments exhibited a markedly reduced potential for horizontal gene transfer. This study highlights that molecular imprinting offers a promising strategy to improve the selectivity of carbon-based photocatalysts toward both antibiotics and associated resistance determinants. The photocatalysis characteristics of antibiotics by carbon-based photocatalysts are clearly summarized in Table 6.

### 4.6. MXene-Based Photocatalysts

MXene, a family of two-dimensional transition metal carbides/nitrides characterized by tunable surface functional groups, exceptional electrical conductivity, and hydrophilic surfaces, has emerged as a highly promising platform for constructing advanced photocatalysts targeting antibiotic degradation [109]. The unique layered structure, abundant exposed active sites, and tailorable electronic properties of MXene facilitate efficient light harvesting and, crucially, promote the spatial separation and transport of photogenerated charge carriers, effectively addressing the key bottlenecks of rapid recombination and sluggish kinetics prevalent in conventional photocatalytic systems [110,111].

Typically synthesized through the selective etching of aluminum layers from MAX phases (e.g., Ti_3_AlC_2_) using hydrofluoric acid (HF) or fluoride-containing solutions, MXenes (predominantly Ti_3_C_2_T_X_) are frequently integrated with semiconductors to form heterostructured composites. Advanced synthesis strategies, including in situ oxidation, hydrothermal/solvothermal assembly, electrostatic self-assembly, and surface functionalization, enable precise architectural control over MXene-based photocatalysts. For instance, a Bi_2_MoO_6_/TiO_2_/Ti_3_C_2_ composite, fabricated via a hydrothermal process (180 °C, 12 h) utilizing Bi(NO_3_)_3_·5H_2_O, Na_2_MoO_4_, and Ti_3_C_2_ precursors, demonstrated remarkable efficiency, degrading 87.5% of TC within 150 min under visible light irradiation. This enhanced performance was ascribed to the Ti_3_C_2_ nanosheets acting as highly efficient electron mediators within a Z-scheme heterojunction, significantly accelerating interfacial charge transfer between Bi_2_MoO_6_ and TiO_2_ while effectively suppressing electron–hole recombination [112]. Similarly, Ag/TiO_2_/Ti_3_C_2_ hybrids were engineered by thermally oxidizing Ti_3_C_2_ in an inert atmosphere (450 °C, Ar) to generate in situ TiO_2_ nanoparticles, followed by the photodeposition of plasmonic Ag nanoparticles (5 wt%). This composite exhibited superior visible-light photocatalytic activity for the degradation of sulfamethazine compared to pristine TiO_2_, attributed to the synergistic interplay between Ag’s LSPR effect, which broadens light absorption into the visible spectrum, and the high conductivity of Ti_3_C_2_, which facilitates rapid electron extraction and transport, thereby minimizing charge recombination and enhancing ROS generation [113].

**Figure 8:**
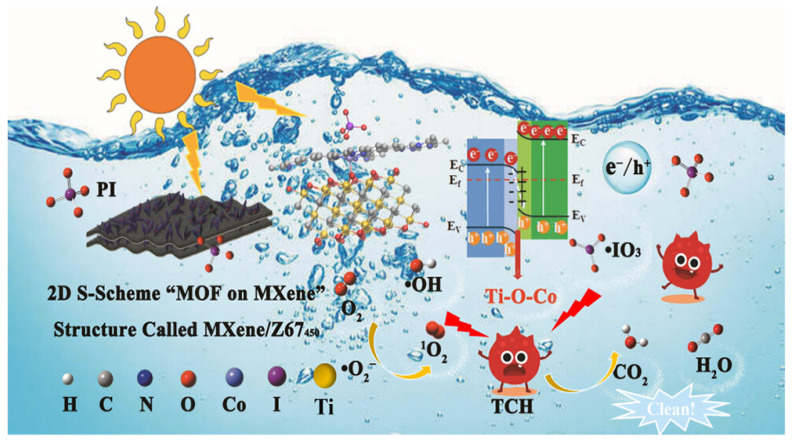
Schematic illustration of the S-scheme MXene/Z67_450_ “MOF-on-MXene” heterostructure for visible-light-driven PI activation and efficient tetracycline hydrochloride degradation [115].

The photocatalytic mechanisms underpinning MXene-based systems are diverse and often involve synergistic effects. Key mechanisms include the formation of Schottky junctions, S-scheme or Z-scheme heterostructures, and the integration of photothermal conversion. For example, in Ti_3_C_2_/Bi_12_O_17_Cl_2_ Schottky heterojunctions synthesized via a one-step solvothermal method, a work function difference establishes an interfacial electric field that drives the transfer of photogenerated electrons from Bi_12_O_17_Cl_2_ to Ti_3_C_2_. This efficient charge separation facilitates the generation of potent ROS (${•O}_{2}^{-}$ and •OH radicals). Optimally, the composite with 3 wt% Ti_3_C_2_ achieved an impressive 95.64% removal of tetracycline TCH within just 60 min under visible light, highlighting the critical role of the Schottky barrier in suppressing recombination and leveraging MXene conductivity [114]. In addition to Schottky-type contacts, constructing MOF-on-MXene S-scheme heterostructures has recently emerged as an effective strategy to exploit both the submetallic conductivity of MXenes and the abundant catalytic sites of MOFs. Chen et al. [115] designed a 2D S-scheme “MOF-on-MXene” photocatalyst (MXene/Z67_450_) by in situ growth and pyrolysis of ZIF-67 on Ti_3_C_2_T_X_, generating interfacial Ti–O–Co and Co–N_4_ bonds that create an internal electric field and atomic-level charge-transfer channels. In a periodate-based advanced oxidation process, the MXene/Z67_450_ heterostructure exhibited markedly enhanced activity for tetracycline hydrochloride degradation, outperforming MXene_450_, Z67_450_ and several commercial metal oxides, owing to more efficient photocarrier separation and facilitated PI adsorption/activation at the S-scheme interface. Spectroscopic analyses, electrochemical measurements and DFT calculations confirmed that the built-in electric field drives photogenerated electrons from Z67_450_ to MXene_450_ through the Ti-O-Co bridge while preserving the strong oxidation capacity of holes in Z67_450_, thus enabling the generation of multiple reactive oxygen and iodine species (e.g., ^1^O_2_, •OH, ${•O}_{2}^{-}$, •IO_4_ and •IO_3_) for selective TCH removal, as schematically illustrated in Figure 8.

Surface modification of MXene provides a novel approach for further tailoring its properties. For instance, sulfonated Ti_3_C_2_ (Ti_3_C_2_-SO_3_H) was coupled with g-C_3_N_4_ via hydrothermal treatment to form a composite. The resulting Schottky junction at the interface significantly reduced charge recombination, enabling 75.4% TC removal under visible light in 120 min. Crucially, the introduced sulfonic acid groups (-SO_3_H) enhanced the surface hydrophilicity of Ti_3_C_2_, improving pollutant adsorption and increasing the accessibility of active sites for ROS-mediated degradation [116]. Beyond charge separation, the inherent photothermal conversion capability of MXenes broadens their utility, particularly under near-infrared (NIR) irradiation. A complex composite, NaYF4: Tm^3+^/Er^3+^/Yb^3+^@BiOI/TiO_2_-Ti_3_C_2_, synthesized via electrostatic self-assembly and calcination, combined upconversion nanoparticles (UCNPs) with MXene-derived TiO_2_ [117]. The layered structure of Ti_3_C_2_ MXenes, the flower-like morphology of TiO_2_@Ti_3_C_2_, and the uniform dispersion of UCNPs within BiOI nanosheets. The TiO_2_ nanoflowers in the TiO_2_@Ti_3_C_2_ composite were uniformly distributed across the Ti_3_C_2_ MXene layers, enhancing the material’s surface area and, consequently, improving photocatalytic efficiency. Furthermore, the UCNPs were evenly dispersed within BiOI, facilitating effective energy transfer from UCNPs to BiOI. The UCNP@BiOI@TiO_2_-Ti_3_C_2_ composite demonstrated the synergistic effects of UCNPs and TiO_2_@Ti_3_C_2_, where the incorporation of TiO_2_-derived Ti_3_C_2_ MXene not only extended the light absorption to the NIR region but also enhanced the photothermal effect, accelerating the reaction rate. These findings indicate that the integration of Ti_3_C_2_ MXenes, UCNPs, and BiOI significantly enhances the photocatalytic performance of the composite by expanding its light spectrum utilization and promoting charge separation.

Plasmonic enhancement can be further integrated into MXene-containing systems to strengthen visible-light harvesting and intensify charge-carrier dynamics. A typical case is a ternary Au/g-C_3_N_4_/Ti_3_C_2_ (AGM) platform used for pharmaceutical degradation (e.g., cefixime). In this composite, Ti_3_C_2_ acts as a metallic, full-spectrum absorber and electron-transport highway, while Au nanoparticles contribute LSPR to broaden visible absorption and supply additional reaction sites. Compared with the binary counterparts, the ternary AGM03 sample exhibited the highest photocatalytic efficiency (64.69% cefixime removal under visible light), which was associated with suppressed radiative recombination (lower photoluminescence), improved photocurrent response, and reduced interfacial charge-transfer resistance. Notably, this system also highlights a practical design constraint: excessive Ti_3_C_2_ loading can reduce apparent light utilization due to its strong black-body absorption and shielding effect, indicating the necessity of optimizing MXene content rather than maximizing it [109]. Beyond “conductive support” behavior, MXenes can participate more actively in constructing interfacial charge-separation motifs, including Schottky-type contacts and multi-junction configurations, Ti_3_C_2_ acted as a powerful structural modulator in the MOF-based system (MIL-125-NH_2_). Its presence during synthesis induced surface reconstruction, resulting in the in situ formation of TiO_2_ and the creation of a dual-heterojunction architecture (NH_2_-MIL-125(Ti)/TiO_2_/Ti_3_C_2_). This unique configuration significantly enhanced the carrier density and facilitated efficient interfacial charge separation and transfer, which significantly boosted both H_2_O_2_ production and TC degradation efficiency. The dual-heterojunction structure, comprising the interaction between TiO_2_ and Ti_3_C_2_, electrons can be extracted and transported more efficiently through Ti3C2, while the coupled TiO2 and MOF domains provide complementary redox sites, collectively improving carrier utilization and photocatalytic kinetics for TC removal, as illustrated in the proposed mechanism for H_2_O_2_ production and TC degradation under visible light illumination [118]. MXenes are also highly effective in facilitating direct Z-scheme mechanisms. In the Bi_2_WO_6_/g-C_3_N_4_/Ti_3_C_2_ composite, Ti_3_C_2_ served as an efficient solid-state electron mediator, accelerating charge transfer between the Z-scheme paired Bi_2_WO_6_ and g-C_3_N_4_ semiconductors. This resulted in substantially improved rates of CIP degradation and, notably, concurrent photocatalytic H_2_ production, demonstrating the potential for integrated pollutant removal and energy recovery [119]. Furthermore, MXenes readily form beneficial Schottky junctions with various semiconductors. In the Ag_2_WO_4_/Ti_3_C_2_ composite, the metallic conductivity and large surface area of Ti_3_C_2_ provided efficient pathways for photogenerated electron transport, extending charge-carrier lifetimes. Importantly, the Ti_3_C_2_ support also enhanced the structural stability and corrosion resistance of the often photocorrosive Ag_2_WO_4_, while enabling effective antibiotic degradation [120]. The photocatalysis characteristics of antibiotics by MXene-based photocatalysts are summarized in Table 7.

### 4.7. Other Photocatalysts

In addition to the widely investigated categories such as metal oxides, bismuth-based materials, silver composites, MOFs, carbon-based systems, and MXenes, several other emerging classes of photocatalysts have also exhibited considerable potential for the degradation of antibiotics. These “other” photocatalysts are characterized by structural diversity, tunable band structures, and unique functionalities, thereby expanding the material toolbox for photocatalytic water remediation.

Perovskite-type and titanate-based photocatalysts have attracted significant attention given their intrinsic ferroelectric polarization and favorable electronic structures, which facilitate charge separation. For example, CuMn_2_O_4_-BaTiO_3_ heterojunction nanocomposites demonstrated almost complete TC degradation within 60 min, achieving a high-rate constant (0.358 min^−1^) and excellent recyclability over five cycles. The enhanced efficiency was attributed to synergistic heterojunction-induced charge transfer and improved light capture by BaTiO_3_ [121]. Similarly, palm-tree-wood-derived activated carbon, functionalized with SrTiO_3_ nanoparticles, effectively degraded cefixime in a continuous-flow system. Optimization of operating parameters such as pH and liquid hourly space velocity further confirmed the practical potential of titanate-based composites for large-scale wastewater treatment [122].

Sulfide-based photocatalysts are another promising category, benefiting from narrow bandgaps and strong visible-light harvesting capacity. However, their photocorrosion and agglomeration issues necessitate structural engineering. A zeolite-supported CoS2/MoS2 heterojunction achieved 96.7% TC removal under visible light, with zeolite enhancing antibiotic adsorption and facilitating intimate contact between pollutant molecules and active sites. The resulting heterojunction significantly improved charge carrier separation and maintained stable performance across multiple cycles [123]. Likewise, an S-scheme Bi_2_WO_6_/CoIn_2_S_4_ heterojunction achieved ~90% TC degradation within 3 h, where band alignment enabled efficient carrier migration and promoted both •OH and ${•O}_{2}^{-}$ radical generation. Toxicity analysis further revealed that most intermediates exhibited reduced ecotoxicity compared with the parent antibiotic, corroborating the environmental advantages of such systems [124].

Polymer- and framework-based photocatalysts represent an emerging frontier, particularly conjugated microporous polymers and covalent triazine frameworks. A 2D/2D heterojunction constructed from covalent triazine frameworks and graphitic carbon nitride nanosheets (CNNS) exhibited 95.8% removal of sulfamethazine within 180 min under solar simulation. The intimate interfacial contact enhanced charge separation and broadened light absorption, while mechanistic studies identified ${•O}_{2}^{-}$ radicals as the dominant active species. This work exemplifies the promise of metal-free photocatalytic systems for the sustainable degradation of antibiotics [125].

Plasmonic and indium oxide-based systems further highlight the versatility of “other” photocatalysts. In this respect, Au-modified In_2_O_3_ nanoparticles achieved 99% removal efficiency of both TC and OFL under visible light, benefiting from the LSPR of the Au nanoparticles. This plasmonic effect enhanced UV–vis absorption and suppressed electron–hole recombination, while radical quenching confirmed the key roles of h^+^ and ${•O}_{2}^{-}$ in the multi-pathway degradation. Importantly, the composites maintained >90% activity across three cycles in various water matrices, substantiating their practical robustness [126]. Similarly, plasmonic Cu-WO_3_ biochar composites achieved 75% TC degradation in 90 min. In this case, the biochar provided abundant adsorption sites, while the Cu nanoparticles induced plasmonic excitation, boosting visible-light utilization and electron–hole separation. Radical scavenging experiments validated that ${•O}_{2}^{-}$ radicals were the primary active species [127].

## 5. Bridging Lab to Field: Sunlight, Real Wastewater, Immobilization and Scalable Systems

Translating photocatalytic antibiotic degradation from proof-of-concept batch tests to deployable water treatment requires that catalysts and reactors be evaluated under (i) realistic irradiation (especially natural sunlight), (ii) complex water matrices, (iii) retrievable/immobilized catalyst formats, and (iv) scalable continuous-flow configurations with defensible energy, toxicity, and stability metrics.

### 5.1. Sunlight-Driven Operation and Realistic Photonic Reporting

Natural sunlight is an attractive energy source because it can drastically reduce operating costs dominated by artificial light inputs, and several reviews explicitly identify sunlight-powered photocatalytic membranes (PMs) as a practical direction for antibiotic removal [128]. However, sunlight introduces variability in intensity, spectral distribution, incident angle, and intermittency, which can cause large differences in apparent kinetics even for “similar” materials and defect strategies [129]. Therefore, for field relevance, studies should report photonic parameters (e.g., spectral irradiance, photon flux, reactor optical path length, illuminated area, solution depth) and normalize performance using metrics such as apparent quantum yield and electrical energy per order [130].

Beyond sunlight, light-emitting diode (LED) photoreactors are increasingly emphasized for scale-up due to wavelength selectivity, lower heat generation, and modular design enabling microreactors, chip/strip reactors, and improved photon utilization relative to conventional lamps. For example, a low-energy Mn–WO_3_ LED photoreactor was assessed with EEO, highlighting the need to compare systems on an energy-normalized basis rather than only pseudo-first-order rate constants [130]. A different device-level strategy is “light delivery engineering”, such as a compact photo-reactor based on luminous textiles (woven optical fibers) that intensifies light distribution and was extrapolated toward pilot-scale operation for antibiotic degradation [131].

### 5.2. Real Wastewater Matrices: Matrix Effects, Deactivation, and Comparability

Performance under ultrapure water often overestimates real-world removal because real matrices contain dissolved organic matter (DOM), inorganic ions, particulates, turbidity, and co-contaminants that can (i) scavenge reactive species, (ii) screen light, (iii) occupy adsorption/active sites, and (iv) promote fouling or catalyst deactivation. A representative example is SMX removal where direct photolysis in real wastewater showed negligible mineralization due to matrix filtering effects, whereas photoelectrocatalysis maintained functionality, illustrating that the “best” approach can be matrix-dependent [132]. Conversely, some systems show limited sensitivity to common anions, so mechanistic conclusions should be validated in multiple representative waters rather than a single synthetic matrix. Real wastewater tests should therefore be stratified by matrix complexity (e.g., secondary effluent, pharmaceutical wastewater, black water, high-salinity brines), and should quantify matrix descriptors such as UV254, TOC/DOC, alkalinity, ionic strength, and turbidity, so that cross-study comparisons and scaling models become meaningful. In black water, for instance, the complex composition increased adsorption load and inhibited photocatalysis on a g-C_3_N_4_/TiO_2_-modified PVDF membrane, even though the same membrane performed better in pure water [133].

### 5.3. Immobilization and Retrievability: From Powders to Membranes, Fibers, and 3D Supports

Powder photocatalysts remain difficult to separate and reuse at scale, which is why immobilization-based strategies are repeatedly highlighted as essential for practical deployment. PMs and photocatalytic membrane reactors (PMRs) directly address this issue by integrating size-exclusion separation with in situ degradation, thereby lowering catalyst loss and potentially shrinking system footprint [128]. A recent PMR-focused review also stresses that PMRs can tackle both chemical pollutants and antimicrobial-resistant bacteria, while noting that membrane stability, light utilization, and fouling control remain decisive bottlenecks.

Many recent PM designs combine improved hydrophilicity/porosity with heterojunction engineering to enhance charge separation while maintaining flux and recyclability. A nitrogen-deficient g-C_3_N_4_-BiOBr-Bi_2_WO_6_/PVDF ultrafiltration membrane achieved high dye retention and exhibited strong flow-circulation visible-light degradation performance (e.g., RhB 97.7% in 1 h, final 99.6%) with self-cleaning behavior. For antibiotic-focused PMs, a PVDF/CeO_2_@GO-COOH membrane combined photodegradation with photo-filtration, delivering high CIP removal and strong reusability with flux recovery rate 97.5% after five cycles [134]. Because fouling is intensified in real waters, PMs increasingly incorporate “self-cleaning” functions, including light-triggered degradation of foulants and surface chemistry that minimizes oil/organic adhesion. A g-C_3_N_4_@MXene composite membrane achieved high permeability and multi-pollutant removal while sustaining multiple cycles without deterioration, demonstrating the potential of 2D/2D architectures for antifouling-compatible water treatment [135]. A particularly field-oriented route is to integrate advanced oxidation chemistry directly into filtration via reactive membranes, e.g., photo-Fenton-like ceramic membranes that combine separation with in-membrane radical production. Such “filtration-plus-AOP” formats can reduce suspended solids while simultaneously degrading antibiotics and mitigating membrane fouling, offering a clearer pathway to continuous operation than batch slurry photocatalysis [136].

### 5.4. Device-Integrated Photocatalysis: From Materials to Deployable Water-Treatment Units

Recent progress increasingly shows that the key bottleneck in photocatalytic antibiotic removal is not only material activity, but also how nanostructured photocatalysts are packaged into retrievable, robust, low-cost devices that operate under natural sunlight and realistic water matrices. A representative example is a sunlight-driven floating photocatalytic device based on BiOBr grown on lightweight expanded clay aggregates (LECA), where the active phase is immobilized on a buoyant, porous support to float at the air-water interface, thereby maximizing both irradiation and surface aeration while avoiding the powder-recovery problem [137]. Importantly, this work went beyond lab batch tests and performed laboratory-scale and real-scale outdoor experiments under natural solar irradiation, demonstrating that the floating BiOBr/LECA composite could fully degrade diclofenac, while ibuprofen removal was more limited and sensitive to water-matrix effects and competitive/deactivation phenomena. Transformation-product tracking further indicated that pollutant structure and pathway preference (e.g., dechlorination-derived products) can strongly influence apparent field performance by gradually occupying active sites, highlighting that device design must be paired with TP/toxicity-aware evaluation rather than parent compound disappearance alone.

From a practical standpoint, such device-oriented demonstrations are essential because they explicitly address real deployment constraints (e.g., catalyst retrieval, stability over reuse cycles, operation without external mixing, and compatibility with variable solar irradiance), which are often missing in slurry-photocatalyst literature. At the same time, the broader environmental context underscores the urgency of scalable, accessible treatment routes, as pharmaceutical consumption and disposal can generate “waves” of drug pollution that stress conventional wastewater infrastructure and increase exposure risks in vulnerable settings [138]. Therefore, this review should systematically include and compare immobilized/floating reactors, photocatalytic membranes, 3D-structured flow-through modules, and other device-integrated platforms as a distinct bridge between nanomaterial innovation and applied water treatment.

## 6. AI-Driven Design and Optimization of Photocatalysts for Enhanced Antibiotic Degradation

**Figure 9:**
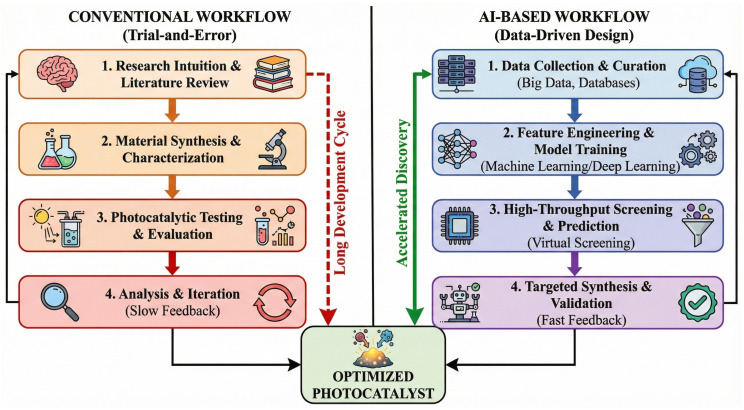
Schematic comparison between the conventional trial-and-error workflow (**left**) and the AI-based data-driven workflow (**right**) for the design and optimization of photocatalysts.

The development of photocatalysts for antibiotic degradation has traditionally relied on iterative synthesis and experimental testing. While these methods have yielded significant progress, they are inherently time- and resource-intensive. Photocatalytic performance depends on multiple interdependent parameters, including light absorption capability, charge carrier dynamics, surface adsorption characteristics, and stability under complex water chemistries. Optimizing such multi-dimensional variables through conventional experimental approaches is challenging. In recent years, artificial intelligence (AI) and machine learning (ML) have emerged as powerful tools for accelerating the design, prediction, and optimization of photocatalytic systems, offering both predictive accuracy and mechanistic interpretability. As illustrated in Figure 9, the AI-based workflow fundamentally accelerates the discovery process by shifting from slow experimental iteration to rapid data-driven prediction, contrasting sharply with the conventional approach. It is now understood that AI-driven strategies provide three major advantages. First, they enable high-throughput virtual screening, narrowing down candidate photocatalysts prior to costly synthesis. Second, they facilitate mechanistic insights by correlating catalyst structure with observed performance, often revealing descriptors such as bandgap energy, surface defect density, or functional group distribution. Third, AI models can predict toxicity evolution during degradation, ensuring not only efficiency but also environmental safety [139]. Collectively, these approaches represent a paradigm shift toward the rational and sustainable design of photocatalysts.

### 6.1. AI Methods for Photocatalyst Design and Mechanistic Insights

AI has emerged as a powerful strategy to accelerate the rational design, prediction, and optimization of photocatalysts for antibiotic degradation. Traditional experimental exploration of photocatalytic materials typically follows a time-consuming “design–synthesis–characterization–evaluation” cycle, where the relationships between microscopic structure, surface chemistry, and macroscopic activity are explored through extensive empirical trials. By contrast, AI can automatically extract quantitative correlations between catalyst features and degradation performance from large datasets, thereby enabling rapid screening of promising materials, guiding defect or dopant engineering, and revealing hidden mechanistic descriptors that control activity and stability [1].

In recent studies, machine-learning models trained on structural and physicochemical descriptors such as band-gap (Eg), conduction- and valence-band potentials, specific surface area, oxygen-vacancy density, and surface functional groups have achieved reliable prediction of apparent rate constants and total organic carbon removal. These approaches allow the rapid identification of optimal electronic and structural features before synthesis. For instance, artificial-neural-network (ANN) analysis of Ni2P–TiO2 catalysts for amoxicillin degradation successfully captured the nonlinear dependence of removal efficiency on irradiation time, catalyst loading, and oxidant dosage, offering data-driven guidelines for process optimization [140]. Similarly, hybrid ML–DFT frameworks have been introduced to link electronic-structure descriptors with experimental reactivity. A representative example is the carbon-dot-implanted SrTiO3 hollow nanosphere system, in which ML and first-principles calculations jointly guided the adjustment of dopant distribution and shell thickness, achieving efficient photocatalysis across a broad pH range while maintaining excellent structural stability [141].

Over the years, a wide range of AI algorithms have been applied to photocatalyst research, each offering unique strengths in prediction, optimization, and mechanistic interpretation. Among these, artificial neural networks (ANNs) have demonstrated efficacy in modeling nonlinear systems. ANN-based models have predicted the degradation efficiency of amoxicillin with Ni_2_P-TiO_2_ catalysts, achieving >95% correlation with experiments while identifying irradiation time and catalyst dosage as key factors. Extending beyond single-target degradation, ANNs were also applied to ZrO_2_-based photocatalysts, accurately simulating both antibiotic mineralization and hydrogen production in hybrid photocatalysis–energy systems. Tree-based algorithms, including random forests and gradient boosting, offer a combination of high predictive accuracy and interpretability for modeling photocatalytic processes. In this respect, gradient boosting models (e.g., XGBoost) have been successfully employed in modeling plasma-assisted antibiotic degradation, achieving strong predictive agreement with experimental outcomes (R^2^ ≈ 0.94). The general ML workflow integrates data collection, model training, performance validation, and interpretability analysis, enabling the identification of gas composition and discharge voltage as the dominant parameters influencing degradation efficiency [142]. Support vector machines (SVMs) are particularly advantageous for smaller datasets and have been combined with density functional theory (DFT) in hybrid frameworks to predict adsorption energies and electronic structures. Such ML-DFT dual approaches have been used to optimize carbon-dot-modified SrTiO_3_ nanospheres, yielding efficient TC degradation across a wide pH spectrum and enhanced catalyst stability against acid–base corrosion.

AI-guided toxicity prediction ensures environmental safety. By coupling ML with quantitative structure–activity relationship (QSAR) models and the Toxicity Estimation Software Tool (TEST), Liu et al. [140] predicted the evolution of toxicity during TC degradation, highlighting that intermediates can sometimes exhibit higher toxicity than parent compounds. Integrating toxicity prediction into photocatalytic modeling thus provides a comprehensive framework for safe water remediation. Collectively, these examples illustrate the power of AI in predicting photocatalytic outcomes, guiding mechanistic interpretation, and ensuring environmental safety.

### 6.2. AI Applications in Photocatalytic Materials and Reactor Systems

Beyond material design, AI has proven valuable for optimizing reactor and process parameters in complex systems. Supervised models trained on multi-variable experimental datasets have been used to predict the degradation efficiency of advanced oxidation systems and to identify the dominant factors controlling reaction kinetics [143]. For example, machine-learning optimization of a microbubble-enhanced cold-plasma reactor established quantitative correlations among gas–liquid contact area, discharge power, and pollutant removal efficiency, reducing experimental workload and guiding operational window selection. In floating photocatalytic devices, predictive models incorporating illumination intensity, flow velocity, and temperature have been able to reproduce degradation trends under variable outdoor conditions, demonstrating how AI can help translate laboratory results to real-world operation [144]. These examples highlight that AI-assisted process modeling is not limited to parameter fitting but can provide physical interpretability and predictive capability for scale-up and continuous-flow design.

AI-driven frameworks have also been applied to multifunctional photocatalysts that integrate adsorption, recognition, and degradation functionalities. A machine-learning-assisted imprinted Ag@PANI/CoFe_2_O_4_/C heterojunction achieved simultaneous enhancement in molecular recognition and photodegradation of tetracycline by automatically selecting structural and compositional parameters governing surface-site distribution [145]. Furthermore, an innovative reverse-synthesis strategy for cobalt-based oxides employed ensemble classifiers to predict catalyst–condition combinations with >90% accuracy, enabling targeted synthesis and validation instead of blind screening [145]. These approaches confirm that AI can efficiently integrate structural optimization, performance prediction, and experimental feedback into a unified “closed-loop” discovery pipeline.

**Figure 10:**
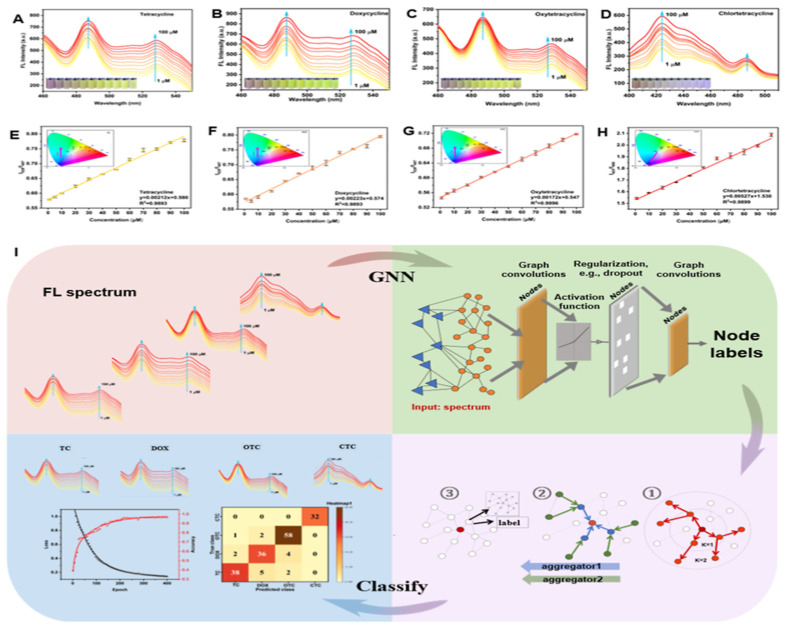
Fluorescence spectra of TC, DOX, OTC, and CTC (**A**–**D**) (1–100 μM); (**E**–**H**) linear relationship between fluorescence ratio; and (**I**) GNNs training flowchart [146].

In recent years, AI-driven approaches have also facilitated the discovery and optimization of advanced photocatalytic materials and engineered systems, bridging the gap between lab-scale innovation and practical implementation. Material level applications include ML-assisted imprinting strategies that enabled the synthesis of Ag@PANI/CoFe_2_O_4_/C heterojunctions with enhanced selectivity and activity. By optimizing imprinting parameters, ML improved the molecular recognition of TC, resulting in superior degradation efficiency and recyclability. Jiang et al. [143] pioneered an innovative reverse synthesis strategy for cobalt-based oxide catalysts, integrating ML with optimization algorithms. By employing the AdaBoost model, a high accuracy (90.57%) was achieved in classifying catalyst performance. SHAP analysis has been applied to reveal the key factors governing degradation efficiency, including reaction time, PMS concentration, and antibiotic molecular fingerprints. Furthermore, the Sparrow Search Algorithm (SSA) was utilized to optimize catalyst selection and experimental parameters for specific antibiotics (LEV, NOR), yielding degradation rates of 94–97%, which were closely aligned with the model’s predictions (error < 2%). Similarly, graph neural networks were applied to MgFe_2_O_4_ nanospheres, enabling simultaneous antibiotic detection, imaging, and degradation within a multifunctional platform [146]. As illustrated in Figure 10, Zhuo et al. constructed a closed-loop environmental health management platform based on spherical MgFe_2_O_4_ nanoparticles that integrate sensing, AI-assisted analysis and photocatalytic remediation of tetracycline antibiotics. In their design, interaction between MgFe_2_O_4_ and four structurally similar TCs (TC, DOX, OTC and CTC) produces distinguishable fluorescence response, which are converted into quantitative concentration readouts via ratiometric calibration curve. These spectral fingerprints are then fed into a GraphSAGE-based graph neural network, whose training workflow—covering data preprocessing, model training/validation and output of classification probabilities. Using 600 spectral samples with a 7:3 training–test split, the GNN achieved ≈91% accuracy in discriminating the four TCs and maintained reliable prediction performance for 180 spiked real samples such as milk and serum. In parallel, the same MgFe_2_O_4_ nanoparticles efficiently activate peroxydisulfate under light irradiation for TC degradation, thus closing the loop from AI-enhanced detection and identification to catalytic removal within a unified nanoplatform. Composite photocatalysts have also benefited from AI-guided optimization. Fe_3_O_4_/g-C_3_N_4_/rGO nanocomposites were optimized through ML combined with response surface methodology, which identified catalyst dosage and irradiation time as critical variables, yielding >85% TC removal under visible light [5].

**Figure 11:**
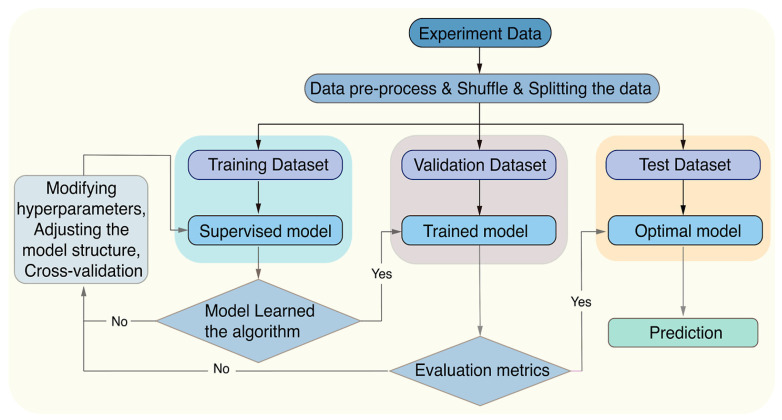
Schematic diagram of the modeling method. Experimental data are processed, shuffled, and split into training, validation, and test datasets. The supervised model is trained using the training data and evaluated with the validation data to obtain a trained model. The trained model is then assessed using the test data, resulting in the optimal model for predicting the DE [147].

Beyond catalyst screening, machine learning has also been employed to optimize operating conditions and reactor configurations for advanced oxidation processes targeting antibiotics. Gao et al. [147] developed a microbubble-enhanced cold plasma activation (MB-CPA) system for the degradation of the sulfonamide antibiotic sulfathiazole and constructed supervised learning models to correlate the degradation efficiency with key operating parameters such as the air-inlet diameter, liquid flow rate and discharge–water distance. As schematically illustrated in Figure 11, experimental data from orthogonal experiments were preprocessed, randomly shuffled and partitioned into training, validation and test sets, followed by comparative training of multiple linear regression and artificial neural network (ANN) models. The ANN markedly outperformed the linear baseline in terms of R^2^ and mean-squared error, enabling accurate prediction of sulfathiazole removal and rapid identification of near-optimal operating windows. Although MB-CPA is a plasma-based advanced oxidation process rather than a conventional semiconductor photocatalyst, this workflow exemplifies how ML-based surrogate models can efficiently explore multi-parameter design spaces and guide the scale-up of photocatalytic and related treatment systems for antibiotic removal. In another study, Bi_4_O_5_I_2_/BCNQDs hydrogels were modeled with ML to predict performance under varying flow and illumination conditions, thereby guiding the design of floating photocatalytic reactors. At the reactor-system level, ML has been used to optimize operating conditions in plasma-assisted photocatalysis. Ensemble models identified ideal discharge voltages and gas ratios that minimized energy consumption while sustaining high antibiotic degradation efficiency [143]. More broadly, ML frameworks have supported the multi-objective optimization of systems balancing degradation efficiency, catalyst stability, and energy costs, advancing practical deployment in real wastewater treatment contexts. These studies highlight how AI can accelerate both material discovery and process optimization, supporting the translation of photocatalysis from controlled laboratory conditions to scalable environmental systems.

## 7. Challenges

While substantial progress has been made in engineering nanomaterials for photocatalytic degradation of antibiotics, several core challenges remain that hinder their real-world implementation. Although numerous materials, ranging from high-specific-surface-area metal oxides and bismuth-based photocatalysts to MXene–semiconductor heterostructures, have demonstrated impressive activity under controlled laboratory conditions, their performance in complex aquatic environments remains poorly understood. In actual wastewater, the coexistence of natural organic matter, anions (e.g., Cl^−^, ${\mathrm{CO}}_{3}^{2-}$), and competing pollutants significantly inhibits ROS generation and catalyst surface accessibility. Moreover, most studies overlook the role of stirring, mass transfer limitations, or long-term structural integrity of the catalyst in such dynamic systems. Although magnetic and hydrophobic modifications have been proposed to facilitate catalyst recovery and dispersion, these approaches often require additional energy input (e.g., continuous stirring) and may compromise sustainability. Optimizing the material interface itself is equally crucial, the synergy between adsorption and photocatalysis is frequently underutilized. While 3D flower-like, hollow, or fibrous nanostructures do enhance surface reactivity and light harvesting, few studies quantitatively examine the interaction between antibiotic molecules and catalyst surfaces under variable pH, ionic strength, or in the presence of coexisting organics. Material composition choices also carry environmental risks, the growing use of metal doping to enhance visible-light responsiveness (e.g., Fe, Cu, Ag) brings concerns about ion leaching and ecological safety. Exploring non-metal dopants, such as boron, nitrogen, sulfur, or phosphorus-may offer more environmentally benign alternatives while preserving bandgap tuning and charge separation benefits. There is also an evident gap in understanding the transformation products formed during photocatalysis. While TOC reduction and parent antibiotic removal are commonly reported, few studies employ comprehensive LC-MS/MS or in silico toxicological prediction to assess the bioactivity, persistence, or ARG propagation potential of intermediate species. Given that partial degradation may lead to even more biologically active or genotoxic byproducts, mechanistic elucidation of degradation pathways is critical.

## 8. Conclusions and Perspectives

In summary, significant strides have been made in the development of advanced nanomaterials for photocatalytic degradation of antibiotics, with the emergence of defect-engineered oxides, bismuth-based heterostructures, plasmonic hybrids, MOF composites, carbonaceous semiconductors, and MXene-based architectures. These materials exhibit diverse morphologies, tunable band structures, and optimized redox pathways, enabling efficient charge separation, broad-spectrum light harvesting, and enhanced ROS generation. Furthermore, the integration of AI has opened new avenues for rational material discovery, mechanistic modeling, and predictive toxicity assessment, marking a paradigm shift toward data driven catalyst design. However, the translation of these laboratory-scale breakthroughs into real world environmental systems remains constrained by several unresolved challenges, including catalyst stability in complex matrices, incomplete mineralization pathways, and secondary pollution from metal leaching or toxic intermediates.

Looking forward, the field must shift from material-centric innovation toward functionally integrated, application-ready systems. One key perspective is the need to embrace contextual complexity-real world water matrices are far more intricate than ideal laboratory systems. The performance of photocatalysts is highly sensitive to ionic competition, dissolved organic matter, pH variability, and mass transport limitations. Therefore, designing photocatalysts with adaptive surface chemistries, such as pH-responsive charge states or zwitterion-targeting interfaces, which may offer a practical route to sustaining reactivity under fluctuating environmental conditions. Moreover, toxicity should no longer be an afterthought. The growing use of nanomaterials in environmental settings demands that intermediate and final degradation products be scrutinized with equal scientific rigor as the parent pollutants. Incorporating LC-MS/MS-based metabolite mapping and in silico toxicological prediction models (e.g., QSAR, TEST) into the standard workflow would allow researchers to evaluate not just degradation efficiency, but also ecological compatibility of photocatalytic processes. From a functional standpoint, future systems should aim to transcend single-target applications. Multifunctionality, such as simultaneous antibiotic removal, heavy metal detoxification, or even solar-to-chemical energy conversion-could enable integrated water purification platforms with higher value-to-cost ratios. Coupled with this, the emergence of AI-guided material discovery calls for models that are not only predictive but also interpretable and mechanistically grounded. ML should be tightly coupled with domain knowledge, enabling closed-loop iteration between virtual screening and experimental feedback. In essence, progress in photocatalytic antibiotic degradation will not be defined solely by higher reaction rates or novel compositions, but by the transition from academic prototypes to resilient, safe, and scalable technologies that meet the chemical and biological complexity of real water environments.

## Figures and Tables

**Table 1:** Physical and chemical properties of principal classes of antibiotics.

Type	Compounds	Molecular Formula	Molecular Mass (g/mol)	Structure	Melting Point (°C)	pK_a_ Values	Log Kow
Tetracyclines (TCs)	Tetracycline (TC)	C_22_H_24_N_2_O_8_	444.43	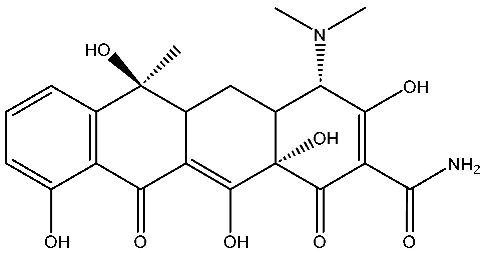	5–170	3.27; 6; 9.612	−1.3
	Oxytetracycline (OTC)	C_22_H_24_N_2_O_9_	460.43	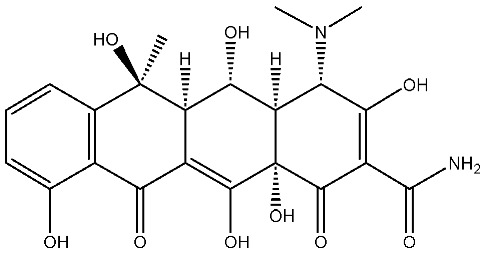	184–185	3.53; 7.25; 9.58	−0.9
	Doxycycline (DOX)	C_21_H_24_N_2_O_8_	1025.89	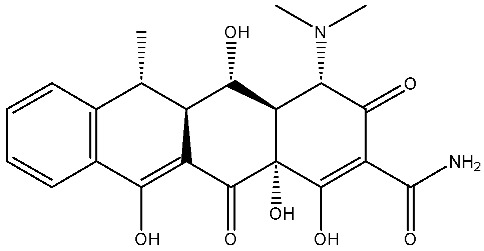	201	30.2; 7.97; 9.15	−0.002
	Chlortetracycline (CTC)	C_22_H_23_ClNO_28_	478.11	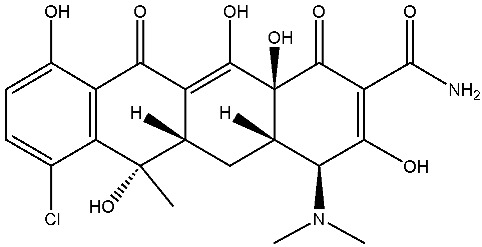	210–215	7.44	−0.62
Sulfonamides (SAs)	Sulfamethoxazole (SMX)	C_10_H_11_N_3_O_3_S	253.28	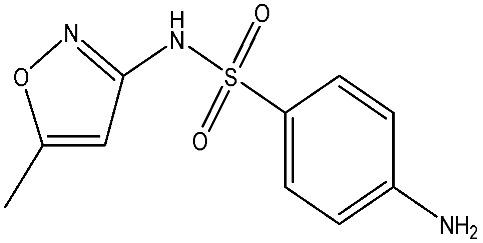	167	1.4; 5.8	0.89
	Sulfamerazine (SMX)	C_11_H_12_N_4_O_2_S	264.30	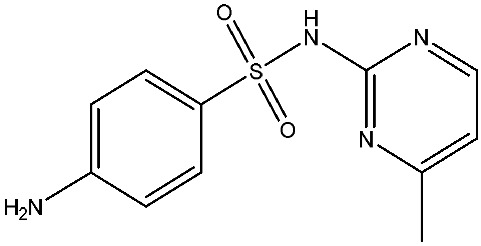	236	2.24; 6.92	1.41
	Sulfadiazine (SDZ)	C_10_H_10_N_4_O_2_S	250.28	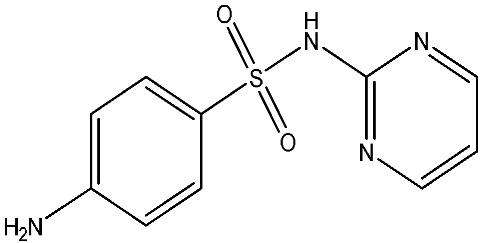	255.5	6.36	−0.0314
Quinolones (FQs)	Ciprofloxacin (CIP)	C_17_H_18_FN_3_O_3_	331.34	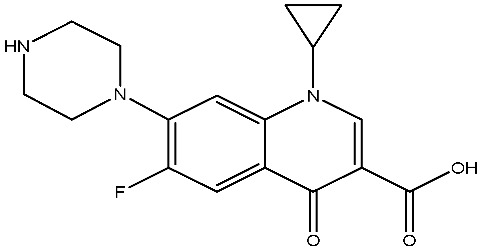	225–257	6.09; 8.74	0.28
	Norfloxacin (NOR)	C_16_H_18_FN_3_O_3_	319.33	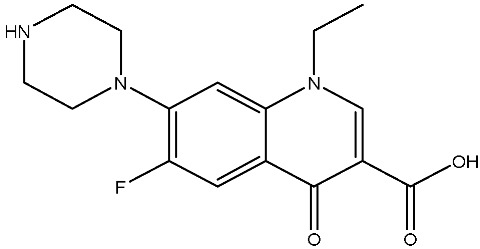	227–228	6.26; 8.85	−1.03
	Enrofloxacin (ENR)	C_21_H_24_FN_3_O_4_	359.39	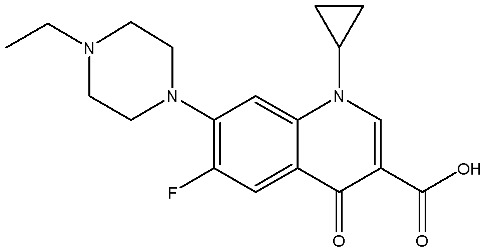	219–221	2.7–3.9	-
	Ofloxacin (OFLX)	C_18_H_20_FN_3_O_4_	361.37	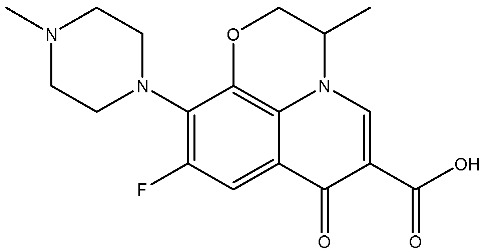	254	5.77; 8.44	−0.39
	Levofloxacin (LEV)	C_18_H_20_FN_3_O_4_	361.37	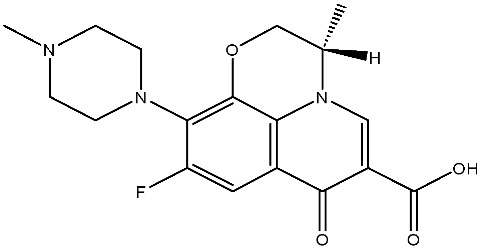	225–227	6.02; 8.05	−0.39
β-lactams	Penicillin G (PEN)	C_16_H_18_N_2_O_4_S	334.39	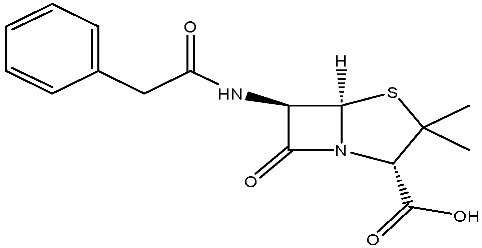	82–83	2.74	-
	Amoxicillin (AMX)	C_16_H_19_N_3_O_5_S	365.40	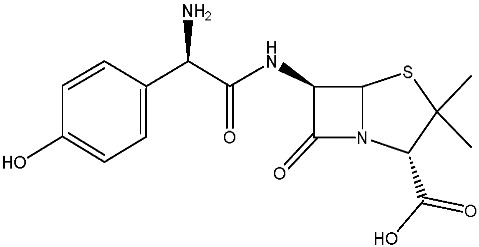	140	3.37; 8.96	0.87

**Table 2:** Photocatalysis characteristics of antibiotic by Metal oxide-based photocatalysts.

Catalyst	Targets	Antibiotic Dosage	Catalyst Dosage	Degradation Efficiency	Light Source	Mian Active Species	Recycle	Ref.
ACT-4	CEF	100 mg/L	1 g/L	99.6% within 240 min	vis	•OH, ${•O}_{2}^{-}$	5	Abdullah et al. [43]
HS-CuFe_2_O_4-σ_	CIP	10 mg/L	0.5 g/L	~100% within 30 min	vis	•OH, HO_2_•/${•O}_{2}^{-}$	5	Ding et al. [40]
Cu_2_O/Bi/Bi_2_MoO_6_	SDZ	10 mg/L	N.P.	98.6% SDZ within 100 min	vis	h^+^, •OH, ${•O}_{2}^{-}$, e^−^	5	Xu et al. [41]
SiO_2_@Fe_2_O_3_@TiO_2_	TC, ENR	10 mg/L (TC), 5 mg/L (ENR)	0.2 g/L	100% TC within 140 min, 100% ENR within 80 min	Natural Sunlight	h^+^, ${•O}_{2}^{-}$	5	Zhang et al. [42]
SA-Fe@ TiO_2_	SMX	10 mg/L	0.3 g/L	94.59% within 30 min	Simulated solar	h^+^, •OH, ${•O}_{2}^{-}$	4	Wang et al. [44]
TiO_2_@Fe_3_O_4_@C–NF	TMP, SGU	5 mg/L	0.67 g/L	80–100% within 8–125 min	UV	h^+^, •OH	7	Yilmaz et al. [46]
CeO_2_NRs	TC	20 mg/L	0.4 g/L	89.35% within 90 min	vis	h^+^, ${•O}_{2}^{-}$	1	Lu et al. [47]
Fc@rGO-ZnO	SMX, CIP	20 mg/L	0.075 g/L	92.55%SMX/95.01% CIP within 180 min	UV	•OH, ${•O}_{2}^{-}$	3	Roy et al. [48]
N-ZnO/C@Bi_2_MoO_6_	SMX	10 mg/L	1.25 g/L	97.3% within 60 min	Vis	h^+^, •OH, ${•O}_{2}^{-}$	4	Wang et al. [49]

N.P.: Not Provided.

**Table 3:** Photocatalysis characteristics of antibiotic by Bismuth-based photocatalysts.

Catalyst	Targets	Antibiotic Dosage	Catalyst Dosage	Degradation Efficiency	Light Source	Mian Active Species	Recycle	Ref.
CZS/CDs/BWO	TC, LEV, NOR, OTC, ENR	20 mg/L	10 g/60 L	TC: 85.2% (40 min); others: 38.9–81.6%	vis	h^+^, •OH, ${•O}_{2}^{-}$	5	Cai et al. [52]
Au@TiO_2_/Bi_2_WO_6_	SMX, TC	15 mg/L	0.5 g/L	96.9% SMX/ 95.0% TC within 75 min	vis	h^+^, •OH, ${•O}_{2}^{-}$	4	Jin et al. [53]
TaON/Bi_2_WO_6_	TC, LEV	20 mg/L	0.2 g/L	93.2% TC/ ~100% LEV within 50–60 min	vis	h^+^, •OH, ${•O}_{2}^{-}$	5	Li et al. [54]
VO-rich BWO	TC	20 mg/L	0.3 g/L	95.12% within 100 min	vis	${•O}_{2}^{-}$	5	Gao et al. [56]
αβ-Bi_2_O_3_/NiAl-LDH	TC, NOR, CIP	15 mg/L (TC); 10 mg/L (CIP, NOR)	1 g/L	96.17% (TC, 120 min); 94.81% (NOR, 180 min); 48.48% (CIP, 180 min)	vis	h^+^, •OH, ${•O}_{2}^{-}$	5	Sun et al. [57]
Nd_0.1_Bi_0.9_VO_4-δ_	CPX	10 mg/L	0.3 g/L	94.3% within 60 min	vis	h^+^, •OH, ${•O}_{2}^{-}$, ClO2	5	Su et al. [58]
rGO/Bi_4_O_5_Br_2_	CIP, NOR, TC	20 mg/L NOR/TC; 10 mg/L CIP	0.5 g/L	80.7% NOR/ 92.5% CIP/ 95.2% TC within 60 min	vis	h^+^, ${•O}_{2}^{-}$	4	Xu et al. [59]
BiVO_4_/O-g-C_3_N_4_	TC	0.3 g/L	0.1 g/L	99.8% within 60 min	vis	^1^O_2_, h^+^, •OH, ${•O}_{2}^{-}$	4	Bao et al. [60]
(Bi)BiOBr/rGO	TC	20 mg/L	1 g/L	>98% within 20 min	vis	h^+^, •OH	5	Jiang et al. [10]
VO-Bi_2_CrO_6_/g–C_3_N_4_	LVFX	15 mg/L	0.9 g/L	92.5% within 120 min	LED	h^+^, •OH, ${•O}_{2}^{-}$	5	Hasanvandian et al. [61]
CQDs/BiOCl/CEM	TC	10 mg/L	0.5 g/L	~98% total removal (30 min dark adsorption + 30 min vis degradation)	vis	h^+^, •OH, ${•O}_{2}^{-}$	4	Zhou et al. [62]
PLS-BiVO_4_/PANI/Ag	FQs	0.025 mmol/L	0.67 g/L	92% FLE/, 95% NOR/95% ENR/, 96% OFL/98% CIP within 120 min	vis	h^+^	5	Gao et al. [63]
Bi_2_Sn_2_O_7_-C_3_N_4_/Y	TC	20 mg/L	1 g/L	80.41% within 90 min	Solar light	h^+^, •OH, ${•O}_{2}^{-}$	4	Heidari, et al. [64]
BiOBr/Cs_x_WO_3_@SiO_2_	TC	20 mg/L	0.5 g/L	93.8% within 60 min	vis	h^+^, •OH, ${•O}_{2}^{-}$	4	Li et al. [65]

**Table 4:** Photocatalysis characteristics of antibiotic by Silver-based photocatalyst.

Catalyst	Targets	Antibiotic Dosage	Catalyst Dosage	Degradation Efficiency	Light Source	Mian Active Species	Recycle	Ref.
Ag_3_PO_4_/PDIsm	TCH	20 mg/L	0.4 g/L	100% within 60 min	vis	h^+^, ${•O}_{2}^{-}$	4	Cai et al. [68]
Ag_3_PO_4_/NiAl-LDH	TCH	20 mg/L	1 g/L	92.8% TCH within 60 min	vis	h^+^, •OH,	4	Fan et al. [69]
Ag/AgBr/AgI@SiO_2_	TC	20 mg/L	0.3 g/L	79.5% within 30 min	vis	${•O}_{2}^{-}$	5	Ma et al. [70]
Ag Ag_3_PO_4_-VAg	SMX	20 mg/L	10 mg	100% within 15 min	vis	•OH, ${•O}_{2}^{-}$	4	Liu et al. [71]
C_3_N_5_/Ag_3_PO_4_	LEV	11.1 mg/L	0.2 g/L	83% LEV within 10–20 min	vis	h^+^, ${•O}_{2}^{-}$	3	Liu et al. [73]
Fe_3_O_4_@mTiO_2_@Ag@GO	FQs	10 g/L	1 g/L	91% NOR within 180 min	UV	•OH, ${•O}_{2}^{-}$	5	Liao et al. [74]
Ag_3_PO_4_/CNT sponge	TC	10 mg/L	1 g/L	90% within 60 min	vis	•OH, ${•O}_{2}^{-}$	5	Jin et al. [75]
(Clay/TiO_2_/Ag0(NPs)	TC, SMX	10.0 mg/L	N.P.	72.4% TC/58.3% SMX within 60 min	vis, UV	•OH	6	Vanlalhmingmawia et al. [4]
Ag/Ag_2_O/C/P/TiO_2_	CIP	10 mg/L	0.15 g/L	89.10% within 120 min	UV	•OH, ${•O}_{2}^{-}$	N.P.	Negoescu et al. [76]

N.P.: Not Provided.

**Table 5:** Photocatalysis characteristics of antibiotic by MOFs-based photocatalysts.

Catalyst	Targets	Antibiotic Dosage	Catalyst Dosage	Degradation Efficiency	Light Source	Mian Active Species	Recycle	Ref.
Bi_2_MoO_6_/UiO66-NH_2_	OFL, CIP	10 mg/L	0.2 g/L	100.0% OFL/96.0% CLP within 90 min	vis	h^+^, ${•O}_{2}^{-}$, •OH	4	Su et al. [81]
NH_2_-MIL-125(Ti)/Ti_3_C_2_ QDs/ZnIn_2_S_4_	TC, SMX	20 mg/L TC; 30 mg/L SMX	0.3 g/L	96% TC within 50 min; 98% SMX within 40 min	vis	${•O}_{2}^{-}$	3	Liu et al. [82]
0.8CuUiO-66	CIP	30 mg/L	0.3 g/L	93% within 60 min	vis	h^+^, ${•O}_{2}^{-}$	5	Yin et al. [83]
SnS_2_@UiO-66	TC	20 mg/L	0.4 g/L	90.0% within 75 min	vis	h^+^, ${•O}_{2}^{-}$	3	Cao et al. [77]
MIL-101(Fe)/Bi_2_WO_6_	TC	20 mg/L	0.2 g/L	82.8% within 60 min	vis	h^+^, ${•O}_{2}^{-}$, •OH	5	Li et al. [80]
Co-ZIF-C_3_N_5_ (10%)	CTC	30 mg/L	0.6 g/L	100% within 6 min	vis	•OH, SO_4_^−^, ${•O}_{2}^{-}$, ^1^O_2_, h^+^	5	Du et al. [84]
CFC/UiO-66-NH_2_/BiOBr	LVFX, CIP	10 mg/L	0.16 g	92.2% LVFX within 120 min; 86.4% CIP within 120 min	vis	h^+^, ${•O}_{2}^{-}$	4	Yu et al. [87]
ZnO@ZIF-8	TC	20 mg/L	0.5 g/L	91% within 50 min	vis	h^+^, ${•O}_{2}^{-}$, H_2_O_2_	5	Zhang et al. [88]
3D-WO_3_-UiO-66@rGO	SMX	20 mg/L	N.P.	90.39% within 60 min	UV	h^+^, ${•O}_{2}^{-}$	10	Huong et al. [90]
ZnFe_2_O_4_/Fe_2_O_3_	CIP	10 mg/L	0.5 g/L	96.5% within 60 min	vis	${•O}_{2}^{-}$, •OH	5	Zhang et al. [91]
ZnO/ZnFe_2_O_4_	TCH	100 mg/L	0.4 g/L	86.3% within 75 min	vis	h^+^, ${•O}_{2}^{-}$	5	Suo et al. [92]
ZnS/ZnIn_2_S_4_	TCH, TC, DOXH, OTC, CTCH	20 mg/L	0.18 g/L	>90% TCH within 60 min; >95% for other TCs within 120 min	Simulated solar	h^+^, ${•O}_{2}^{-}$, •OH	4	Li et al. [14]

**Table 6:** Photocatalysis characteristics of antibiotic by Carbon-based photocatalysts.

Catalyst	Targets	Antibiotic Dosage	Catalyst Dosage	Degradation Efficiency	Light Source	Mian Active Species	Recycle	Ref.
Cu_2_O/RGO/BiVO_4_	TC	100 mg/L	0.5 g/L	96% within 180 min	vis	h^+^, ${•O}_{2}^{-}$, •OH	5	Shen et al. [96]
rGO-ZnS-CuS	OFL	14.4 mg/L	0.2 g/L	~100% within 90 min	vis	•OH, ${•O}_{2}^{-}$	5+	Mahalingam et al. [97]
Ce_5_-CdS/N-rGO_20_	TC	20 mg/L	0.25 g/L	94.5% within 60 min	vis	h^+^, ${•O}_{2}^{-}$, •OH, ^1^O_2_	4	Wang et al. [98]
BCT30	TC	50 mg/L	0.2 g/L	95% within 120 min	Simulated solar	h^+^, •OH	5	Zhang et al. [99]
CoFe-LDH@PBC800	SMX	20–100 μM	0.05–0.5 g/L	100% within 100 min	UV	h^+^, ${•O}_{2}^{-}$, •OH	3	Li et al. [100]
Ag@BC-rGO	RIF	80 mg/L	1–3 mg	N.P.	Scattered sunlight	•OH, ${•O}_{2}^{-}$	N.P.	Saikia et al. [101]
CaIn_2_S_4_-ZnO/Biochar	TCH	50 mg/L	1 g/L	>95% within 60 min	vis	•OH, ${•O}_{2}^{-}$	6	Khlifi et al. [102]
A-CN	TC	20 mg/L	0.05–0.4 g/L	95% degradation, 88% mineralization within 60 min	vis	h^+^, ${•O}_{2}^{-}$	4+	Li et al. [103]
CMCN2	TC	20 mg/L	0.6 g/L	98% within 60 min	vis	h^+^, ${•O}_{2}^{-}$, •OH, ^1^O_2_	4	Zhou et al. [104]
Mo/Nv-TCN	TC	10 mg/L	1 g/L	94.45% within 60 min	vis	h^+^, ${•O}_{2}^{-}$, •OH, ^1^O_2_	4	Zhang et al. [105]
CNNS/NH_4_V_4_O_10_	CIP	10 mg/L	0.5 g/L	92% within 100 min	Simulated sunlight	h^+^, ${•O}_{2}^{-}$, •OH, e^−^	5	Ma et al. [106]
3B-PCN	CIP	10 mg/L	0.4 g/L	87.56% within 60 min	vis	h^+^, •O, •OH	5	Zhang et al. [107]

N.P.: Not Provided.

**Table 7:** Photocatalysis characteristics of antibiotic by MXene-based photocatalysts.

Catalyst	Targets	Antibiotic Dosage	Catalyst Dosage	Degradation Efficiency	Light Source	Mian Active Species	Recycle	Ref.
Bi_2_MoO_6_/TiO_2_/Ti_3_C_2_	TC	10 mg/L	N.P.	87.54% within 150 min	vis	${•O}_{2}^{-}$	4	Qi et al. [112]
Ag/TiO_2_/Ti_3_C_2_	SFM	N.P.	0.1 g/50 mL	~99% within 60 min	Simulated solar light	h^+^, •OH	5	Lee et al. [113]
Ti_3_C_2_/Bi_12_O_17_Cl_2_	TCH	20 mg/L	0.3 g/L	>90% within 60 min	vis	•OH, ${•O}_{2}^{-}$	3	Liu et al. [114]
Ti_3_C_2_–SO_3_H/g-C_3_N_4_	TC	10 mg/L	0.5 g/L	75.42% within 120 min	vis	•OH, ${•O}_{2}^{-}$	5	Zhang et al. [116]
TiO_2_@Ti_3_C_2_/UCNPs@BiOI	TC	40 mg/L	0.25 g/L	90% within 120 min	Scattered sunlight	h^+^, ${•O}_{2}^{-}$	3	Hosseini et al. [117]
Au/GCN/MXene	CEF	5 × 10^−5^ M (~20 mg/L)	20 g/50 L	64.69% within 105 min	vis	h^+^, ${•O}_{2}^{-}$, •OH	3	Kumar et al. [109]
NH_2_-MIL-125(Ti)/TiO_2_/Ti_3_C_2_	TC	N.P.	N.P.	82.80% within 60 min	vis	h^+^, •OH	4	Wu et al. [118]
Bi_2_W_6_/C_3_N_4_/Ti_3_C_2_	CIP	10 mg/L	0.15 g/L	92% within 60 min	vis	h^+^, ${•O}_{2}^{-}$	3	Wu et al. [119]
Ag_2_WO_4_/Ti_3_C_2_	TC, SFE	20 mg/L	1 g/L	62.9% (TC)/88.6% (SFE) within 40 min	vis	h^+^, ${•O}_{2}^{-}$	3	Fang et al. [120]

N.P.: Not Provided.

## Data Availability

No new data were created or analyzed in this study.
